# AI‐assisted design of a VEGFR2 agonistic peptide that promotes angiogenesis and wound repair

**DOI:** 10.1002/pro.70529

**Published:** 2026-03-12

**Authors:** Farzana Yasmeen, Rajath Ramachandran, Rameez Hassan Pirzada, Bogeum Choi, Hana Seo, Wook Kim, Moon Suk Kim, Sangdun Choi

**Affiliations:** ^1^ Department of Molecular Science and Technology Ajou University Suwon Republic of Korea; ^2^ S&K Therapeutics Suwon Republic of Korea

**Keywords:** angiogenesis, molecular modeling, peptide therapeutics, VEGFR2 agonist, wound repair

## Abstract

Vascular endothelial growth factor (VEGF) serves as a principal regulator of neovascularization and is essential for vascular regeneration. In this study, we identified and evaluated VEGF Mimetic Peptide3 (VMP3), a novel synthetic peptide developed as a potential VEGFR‐2/VEGF‐A modulator. VMP3 was generated using an AI‐assisted peptide design framework incorporating long short‐term memory (LSTM) networks, which enabled learning of sequence‐level patterns within known VEGF agonist peptides and assisted the motif‐guided and structure‐informed design of new candidates. Molecular dynamics simulations suggested a stable interaction profile between VMP3 and VEGFR2, supporting the feasibility of receptor engagement. Experimental validation was performed using in vitro, ex vivo, and in vivo methods. In vitro functional assays showed that VMP3 promoted endothelial cell proliferation, migration, and invasion in a manner similar to that of native VEGF. Ex vivo aortic ring assays supported these findings, demonstrating micro vessel sprouting following VMP3 treatment. An in vivo Matrigel plug assay in C57BL/6J mice revealed increased neovascularization following VMP3 treatment, as confirmed by histological and immunohistochemical analyses (H&E and CD31 staining). In addition, in a diabetic mouse wound healing model, VMP3 significantly accelerated wound closure, and tissue‐level analysis of wound sites confirmed enhanced re‐epithelialization and neovascularization, as evidenced by Masson's trichrome, Picrosirius Red, H&E, and CD31 staining. Collectively, these results suggested that VMP3 functions in a manner similar to that of VEGF, promoting angiogenesis.

## 1 INTRODUCTION

Angiogenesis, the formation of new blood vessels, is essential for tissue regeneration, wound healing, and embryonic development (Carmeliet & Jain, 2011; Kretschmer et al., 2021; Papetti & Herman, 2002). Vascular endothelial growth factor (VEGF) is central to this process, exerting its effects primarily through receptor tyrosine kinases, especially VEGFR2. Activation of VEGFR2 triggers downstream signaling cascades, such as the PI3K/Akt and Ras–Raf–MEK–ERK pathways, which promote endothelial cell (EC) proliferation, migration, and survival (Cébe‐Suarez et al., 2006; Gille et al., 2001; Meyer et al., 1999; Otrock et al., 2007). In addition to VEGFR2, VEGF interacts with VEGFR1 and VEGFR3. VEGFR1 primarily functions as a decoy receptor, modulating VEGF bioavailability, while VEGFR3 is essential for lymphangiogenesis and vascular remodeling (Shibuya, 2011; Tammela & Alitalo, 2010). Together, these receptors orchestrate vascular development and homeostasis, integrating pro and anti‐angiogenic signals to maintain vascular architecture (Claesson‐Welsh & Welsh, 2013; Hicklin & Ellis, 2005; Kendall & Thomas, 1993).

VEGF driven angiogenesis proceeds via two principal mechanisms: sprouting angiogenesis, in which ECs form new branches, and intussusceptive angiogenesis, a process that remodels existing vessels (Testa et al., 2008). These mechanisms are tightly regulated by environmental cues, including oxygen availability, inflammatory mediators, and mechanical forces, ensuring appropriate vascular adaptation to tissue demands (Rivilis et al., 2002). Dysregulation of angiogenesis contributes to the pathogenesis of cancer, ischemic diseases, and diabetic retinopathy, as aberrant vascular growth drives disease progression (Crawford et al., 2009; Deveza et al., 2012; Khurana et al., 2005). Given the central role of VEGF in vascular homeostasis, therapeutic modulation of angiogenesis has emerged as a promising strategy for conditions where impaired blood supply leads to tissue dysfunction (Ware & Simons, 1997). Therefore, evaluating the angiogenic potential of novel VEGF mimetic peptides is critical for developing therapies aimed at restoring vascular function in disease settings.

Regulation of VEGF signaling offers significant therapeutic potential for vascular diseases such as atherosclerosis, cardiovascular ischemia, and chronic wounds (Ferrara & Adamis, 2016). Targeting VEGFR2 is of particular interest since it mediates most VEGF induced angiogenic effects (Fallah et al., 2019; Shibuya, 2013). Despite advances in this field, clinical translation of VEGF based therapies faces persistent challenges, including the short half‐life and off target effects of therapeutic agents, as well as difficulties in controlled delivery (Carmeliet & Jain, 2000; Khachigian et al., 2023). While gene and protein therapies have demonstrated some promise, their clinical application is limited by difficulties in achieving precise control of angiogenic activity, highlighting the need for alternative strategies that retain the beneficial properties of VEGF but mitigate its limitations (Fujii et al., 2009).

To address these limitations, research has increasingly focused on developing VEGF mimetics and synthetic molecules that selectively engage VEGFR2 and offer improved stability and bioavailability (Martino et al., 2011). Advances in computational modeling have facilitated the rational design of bioactive peptides with optimized receptor binding properties (Wan et al., 2022). LSTM networks are general sequence models capable of learning patterns from biological sequences; in the present study, this approach was applied specifically to short VEGF‐mimetic peptide sequences to identify and generate candidate bioactive peptides.

Recent artificial intelligence and machine learning approaches, including long short‐term memory (LSTM) networks, have shown promise in learning sequence‐level patterns from biological data when applied to well‐defined peptide families (Ahmad et al., 2023; Gers et al., 2000). In the context of VEGF mimetic design, LSTM‐based models can be used in an AI‐assisted manner to explore sequence variability, identify conserved motifs, and support the rational generation of candidate peptides that resemble VEGF's receptor‐binding features. Such engineered peptides aim to enhance angiogenesis with improved stability, reduced immunogenicity, and tunable receptor activation (Lai et al., 2025).

The integration of AI‐assisted sequence modeling with structure‐guided computational refinement and experimental validation has accelerated the development of next‐generation VEGF mimetic peptides (Geist et al., 2024). By optimizing receptor interactions, these molecules offer controlled and effective regulation of angiogenesis and hold promise as therapeutic alternatives for vascular diseases (D'Andrea et al., 2005, 2017; De Rosa et al., 2016) (Galiano et al., 2004). Notably, despite growing interest in machine learning for peptide engineering, relatively few studies have applied AI‐assisted approaches to the design of VEGF mimetic peptides with demonstrated angiogenic activity and favorable stability profiles, highlighting the relevance of the present work (Di Stasi et al., 2023).

In this study, we describe the design and functional evaluation of a VEGF‐mimetic peptide targeting VEGFR2 signaling. Angiogenic activity was examined using complementary in vitro, ex vivo, and in vivo models, followed by assessment in a diabetic wound healing model to explore therapeutic relevance. Collectively, these experiments establish the peptide's ability to promote vascular responses and tissue repair.

## 2 RESULTS AND DISCUSSION

### 2.1 Peptide design and structural evaluation

Six candidate peptides (VMP1–VMP6) were generated using an AI‐assisted LSTM‐based sequence modeling pipeline and subsequently refined through MOE‐based structural assessment and docking feasibility analysis. All peptides satisfied predefined physicochemical criteria and were synthesized and evaluated in identical in vitro assays. While multiple candidates met the *in‐silico* selection criteria, only VMP3 reproducibly induced angiogenic responses across assays, whereas the remaining peptides did not exhibit significant VEGFR2 activation activity under the tested conditions. Based on these outcomes, VMP3 was prioritized for detailed structural modeling, molecular dynamics (MD) simulations, and downstream biological characterization (Supplementary Table S1). Future studies will include benchmarking against random, motif‐based, and scrambled peptide controls to further quantify the contribution of AI‐assisted sequence design.

**FIGURE 1:**
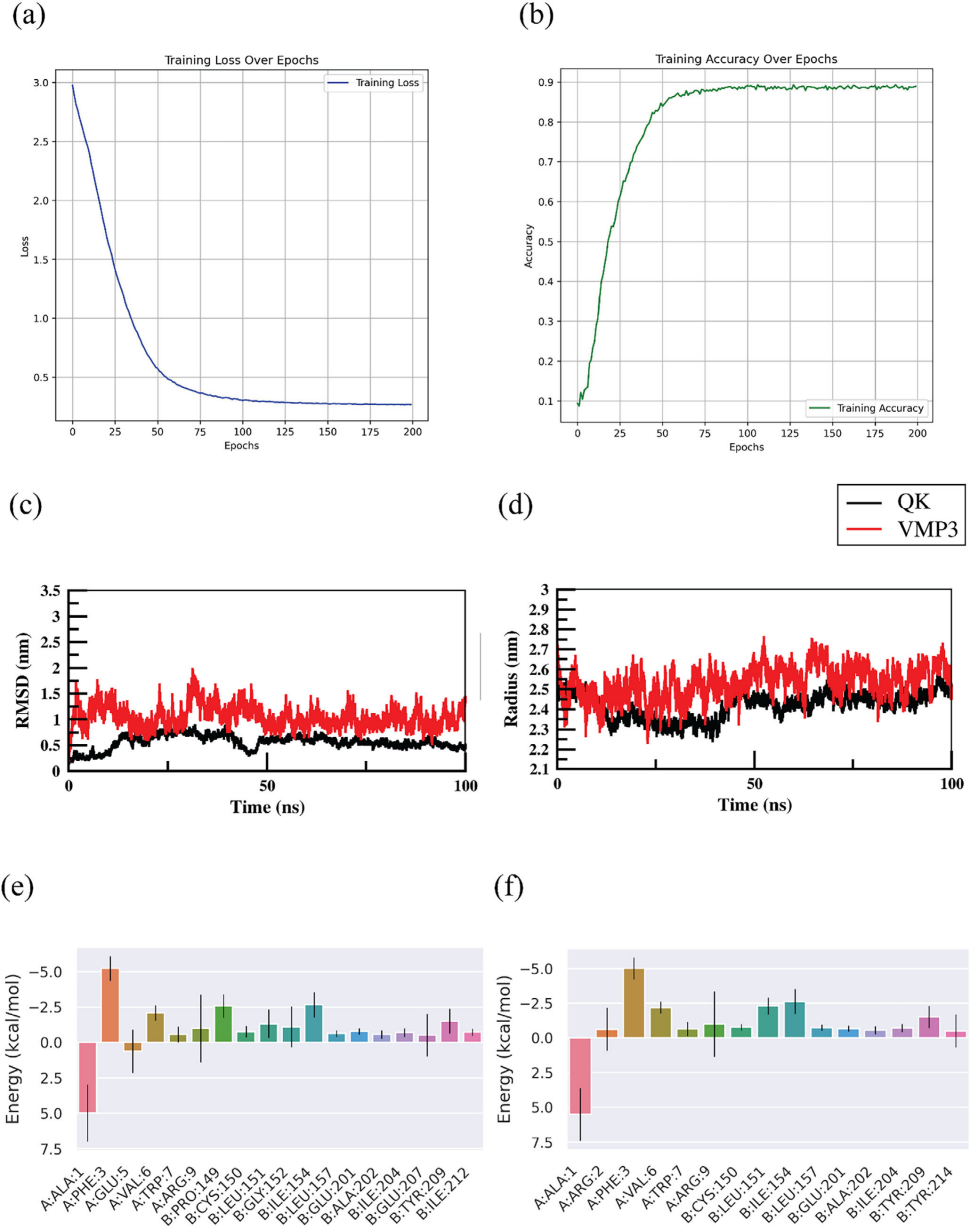
LSTM‐based design and structural stability analysis of the VEGF‐mimetic peptide VMP3. (a) Training loss of the LSTM model over 200 epochs, showing a consistent decrease and convergence. (b) Accuracy stabilizes above 90% after 50 epochs. (c) RMSD analysis of VMP3 and QK during simulations. (d) Radius of gyration (Rg) showing peptide compactness and stability. (e) Total residue contributions and (f) side chain contributions to binding. Chain A and B represent VMP3 and VEGFR2 respectively.

The LSTM model was trained over 200 epochs to generate VEGF‐mimetic peptide sequences. Model convergence was monitored using training loss and accuracy curves (Figure 1a,b), with accuracy exceeding 90% after approximately 50 epochs. This training behavior indicates effective learning of sequence‐level patterns within a constrained VEGF‐mimetic peptide space rather than direct prediction of biological activity, consistent with the intended role of the LSTM as an AI‐assisted sequence pattern generator. Based on its sequence optimization and preservation of key physicochemical and amphipathic features characteristic of VEGF‐mimetic peptides such as QK. QK is a synthetic 15‐amino‐acid VEGF‐mimetic peptide (Ac‐KLTWQELYQLKYKGI‐NH_2_) derived from the receptor‐binding α‐helical region of VEGF‐A and was used as a positive peptide control. It replicates the pro‐angiogenic functions of native VEGF while offering greater stability and lower production costs (Finetti et al., 2012). The stable *α*‐helical structure of the QK peptide is essential for the recognition and activation of the VEGFR2 receptor. Research indicates that the sequence can be modified to enhance receptor affinity, which has been shown to promote vascularization, bone regeneration, and neural tissue repair (D'Andrea et al., 2005; Santulli et al., 2009). VMP3 retained physicochemical properties similar to QK (net charge +2, isoelectric point ~9.3), supporting its potential as a bioactive VEGF mimetic.

The extracellular receptor domain consists of 7 Ig‐homology domains; domains 2 and 3 (D2 and D3) which represent the ligand‐binding domains of VEGFR2. These critical sites were targeted for docking and further advanced to MD simulation. For MD simulation, we used CHARMM36‐modified TIP3P water model and the system consists of 36,099 water molecules and it was neutralized by 8 chloride ions. All the MD parameters were included in the Supplementary Table S2. MD simulations demonstrated that VMP3 maintained root‐mean‐square deviation (RMSD) values in between 0.5 and 1.9 nm, following an initial equilibration period, likely due to conformational differences. QK exhibited relatively lesser RMSD values (0.1–0.8 nm), defining its structural stability, but both peptides remained stable throughout simulations with RMSD below 2.0 nm (Figure 1c). These results indicate that the LSTM‐generated peptide VMP3 achieves structural stability comparable to that of an established VEGF mimetic, supporting the feasibility of AI‐assisted peptide generation followed by structural refinement. Further analysis of the radius of gyration (Rg) confirmed comparable compactness between VMP3 (2.3–2.7 nm) and QK (2.3–2.5 nm), with overlapping gyration patterns observed over the simulation timeframe (Figure 1d).

The binding affinity and key interaction residues of VMP3 with VEGFR2 were evaluated by analyzing final 50 ns (from one of the triplicates, gen_seed = 300, time step = 300 ns) using MM/PBSA calculations. The detailed binding energy calculation criteria is provided in the Supplementary Table S3. The average total binding energy of −27.12 ± 4.14 kcal/mol (mean ± SD over 3 independent simulations, *n* = 1000 frames), indicated strong and favorable interactions, predominantly driven by van der Waals and electrostatic forces. Per‐residue energy decomposition revealed that PHE3 and VAL6, identified previously by AI‐assisted sequence optimization, as principal contributions to receptor engagement by mediating hydrophobic and π–cation interactions. (Figure 1e,f).

Collectively, these results demonstrate that the AI‐assisted LSTM‐based design strategy, when integrated with physicochemical filtering and structure‐guided refinement, successfully yielded a stable and strongly interacting VEGF‐mimetic peptide. While this AI‐assisted design strategy successfully yielded a biologically active VEGF‐mimetic peptide, direct benchmarking against randomly generated, motif‐only, or scrambled peptide sequences was beyond the scope of the present study and will be addressed in future work to further quantify the specific contribution of AI‐assisted sequence modeling; taken together, VMP3's properties in terms of sequence, structure, and predicted binding support its further experimental evaluation as a potential VEGFR2/VEGF‐A agonist.

### 2.2 Thermodynamic insights into the interaction between VMP3 and VEGFR2

**FIGURE 2:**
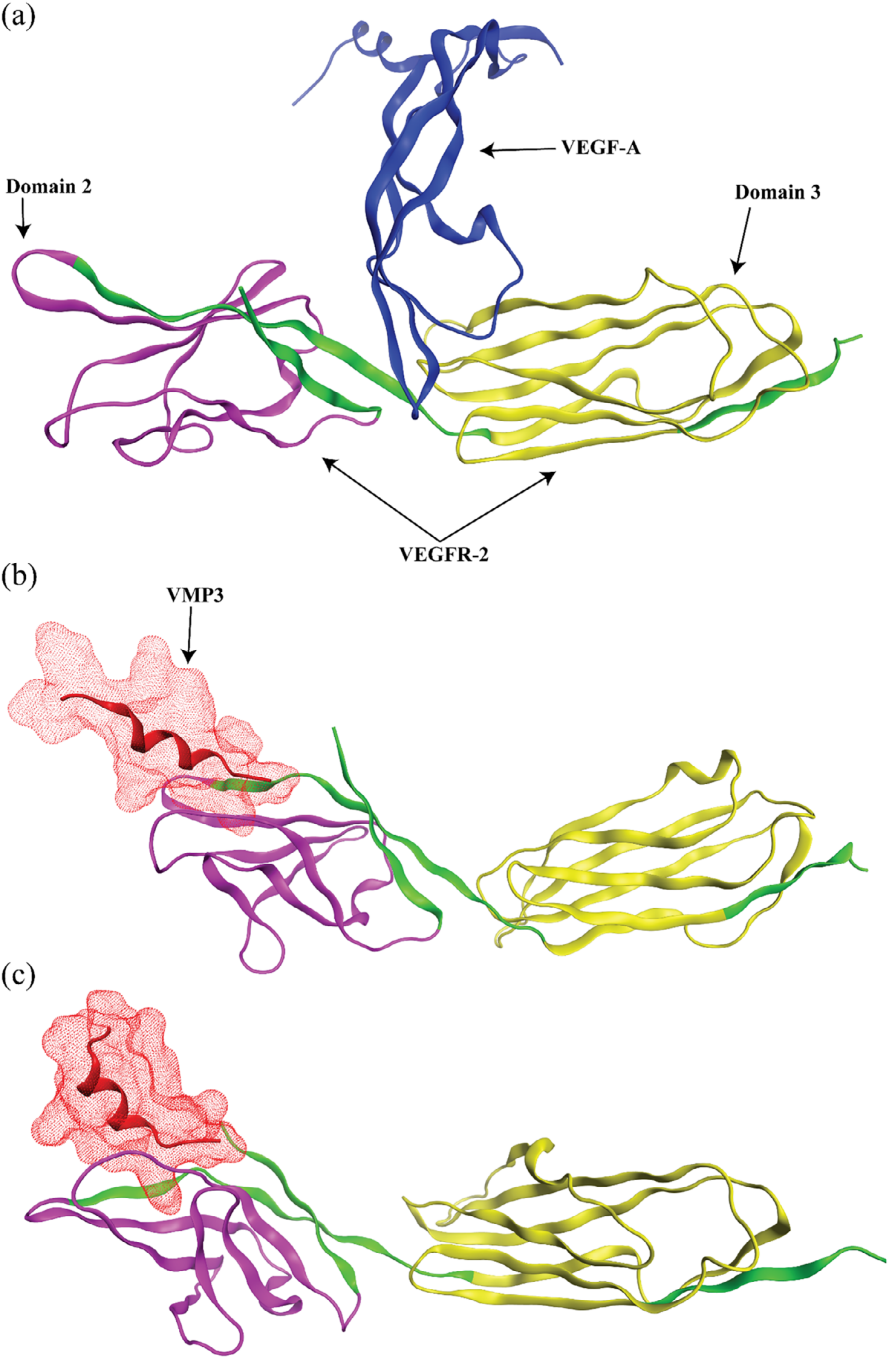
Structural modeling of VEGFR2‐VMP3 interactions and simulation dynamics. (a) Crystal structure of VEGF‐A (blue) bound to VEGFR2 extracellular domains D2 (magenta) and D3 (yellow) (PDB ID: 3V2A), illustrating the canonical ligand‐binding interface. (b) Docked pose of VEGFR2‐VMP3 complex nearby the immunoglobulin‐like domain 2 (D2) site. (c) Final simulation structure sampled at 300 ns.

The crystal structure of VEGF‐A bound to the extracellular domain of VEGFR2 (PDB ID: 3V2A) was used as a structural reference to define the canonical ligand‐binding conformation (Figure 2a). Molecular docking revealed a favorable binding of VMP3 near the immunoglobulin‐like D2 domain of VEGFR2 (Figure 2b). To rigorously evaluate the stability and dynamics of the VEGFR2‐VMP3 complex, triplicate MD simulations were performed, each initiated with distinct random velocity seeds (gen_seed = 100, 200, 300) and extended to 300 ns. The simulations consistently demonstrated stable binding kinetics and comparable thermodynamic behavior, as reflected by overlapping RMSD trajectories and radius of gyration (Rg) profiles across all three replicates (Figure S1). The significant structural rearrangements causing drastic hydrogen bond profiles in all three independent md runs specifically, in all cases after 250 ns the average hydrogen bonds were increased and consistent around 3–4 hydrogen bonds, up to a maximum of 5 hydrogen bonds at the end of simulation. These findings indicate that VMP3 achieves conformational stability at the VEGFR2‐D2 binding interface.

Our simulations suggest that VMP3 binding induces conformational perturbations within the VEGFR2 monomer that propagate across receptor domains, initiating domain reorganization events that modulate the binding pocket architecture within the canonical VEGF‐A recognition site at the D2 and D3 interface as shown in Figure 2c. These structural modifications, characterized by increased pocket depth and optimized electrostatic complementarity, are consistent with enhanced ligand–receptor interaction energetics and may favor conformational states compatible with receptor–receptor interactions, rather than directly promoting spontaneous receptor dimerization through reduced inter‐domain distances and reorientation of transmembrane helices (Figure 2c). Simultaneous stabilization of the D2 domain and conformational rearrangements in domain 3 resulted in a more compact receptor conformation compared to the docked structure with an overall RMSD of 1.92 (Figure S2) and the initial refined crystal structure (RMSD 8.41) (Figure S3). Additionally, we performed triplicate simulations for the apo form (VEGFR2) for 300 ns and superimposed its final structure with the VMP3 bound structure. The subsequent analysis verified that the D3 underwent serious conformational changes upon VMP3 binding with an RMSD of 2.52 Å compared to the final apo form structure (Figure S4). Similarly, these variations were evident throughout the simulation (Figure S5).

The putative allosteric binding site of VMP3 at domain 2 was validated through site‐directed mutagenesis (Alanine scan) by mutating the critical energetically favorable residues of VEGFR2 (ILE154 and TYR209). The point mutation at ILE154 caused total diminishment of critical binding energy while the mutation TYR209 induced significant loss of binding energy favorable for the binding of VMP3 (Supplementary Table S4) and thus supported our proposed binding mechanism. The observed allosteric coupling between the VMP3 binding site and the natural ligand binding domain represents a classic example of positive cooperative binding, where peptide occupancy at the allosteric site increases the apparent binding affinity for VEGF‐A through energetically favorable conformational selection mechanisms (Figure 2).

The crystal structures of complexes between VEGF‐A with D2 and D3, which revealed comparable binding surfaces and similar interactions between the ligands and the receptor but showed variation in D2 and D3 twist angles. The energetically unfavorable homotypic interactions in D4–D7 may be required for re‐orientation of receptor monomers, and this proposed mechanism might prevent ligand‐independent activation of VEGFR‐2 to evade the deleterious consequences for blood and lymph vessel homeostasis arising from inappropriate receptor activation (Brozzo et al., 2012). The mechanism involves the reorientation of receptor domains that makes clarity of computational findings with higher RMSD values and overlaps with our in vitro and in vivo experiments.

### 2.3 Selection of VMP3 as the lead VEGF mimetic peptide

**FIGURE 3:**
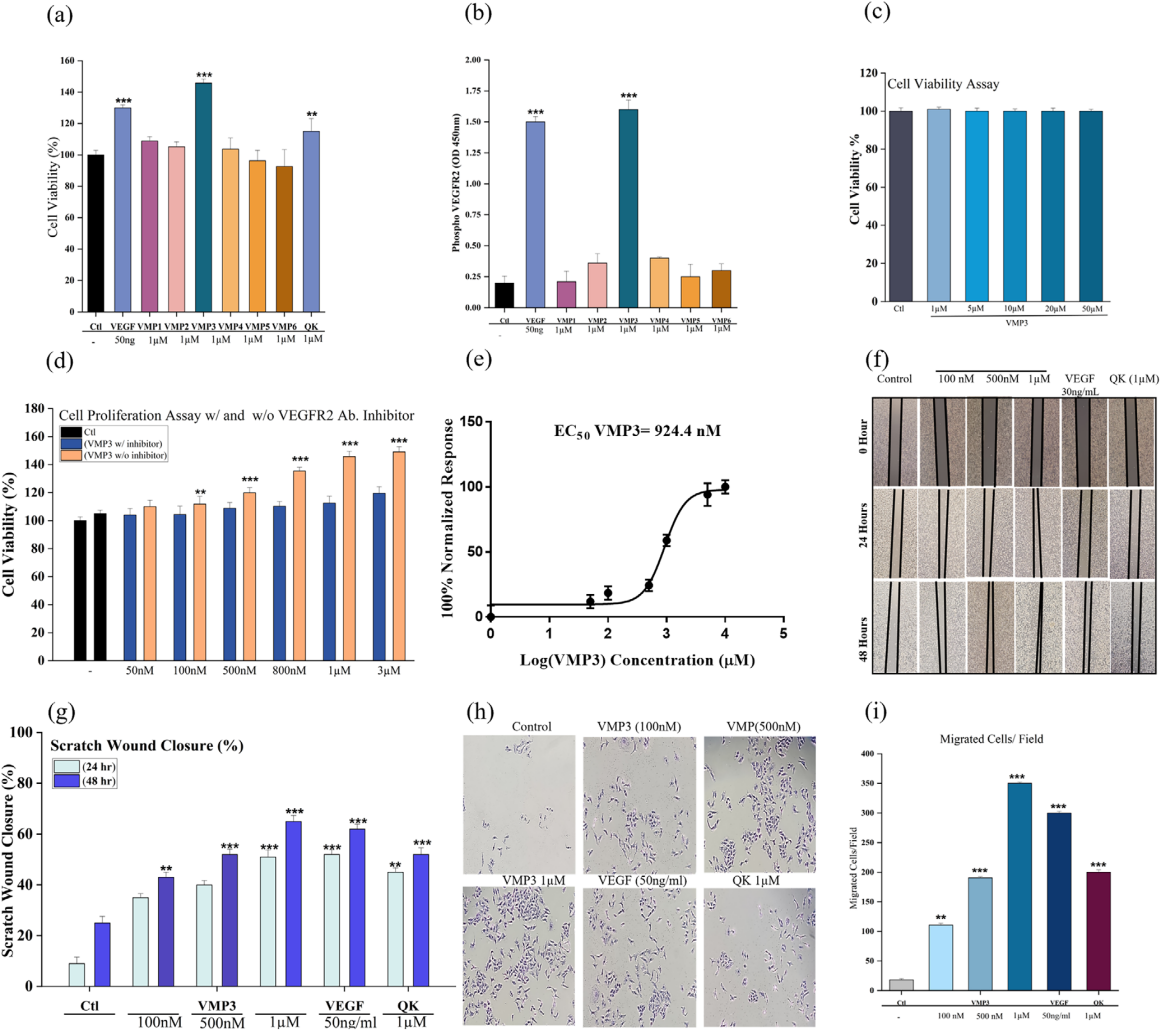
Functional validation of VMP3‐induced angiogenic activity in endothelial cells. (a) VMP3‐induced proliferation of HUVECs assessed by Trypan Blue exclusion assay. (b) PathScan ELISA showing VEGFR2 activation by VMP3. (c) Cell viability assay for VMP3 cytotoxicity. (d) Proliferation with or without VEGFR2 inhibitor. (e) Dose–response curve and EC_50_. (f) Scratch wound images at 0, 24, and 48 h for each group. (g) Wound closure quantification. (h) Representative transwell migration images. (i) Migrated cell quantification. Bars represent mean ± SD (*n* = 3 independent experiments). *p <0.05, **p <0.01, ***p <0.001 versus negative control (two tailed unpaired Student's *t*‐test), analyzed using GraphPad Prism v8 (La Jolla, CA, USA).

To identify the most effective VEGF mimicking peptide, two complementary approaches were employed: a cell proliferation assay to assess functional activity and a VEGFR2 PathScan ELISA to evaluate VEGFR2 phosphorylation. All six candidate peptides were first assessed for pro‐proliferative activity under serum‐starved conditions using the Trypan Blue exclusion assay, which directly quantifies live cell numbers (Figure 3a). In parallel, all peptides were evaluated for their ability to induce VEGFR2 phosphorylation using the VEGFR2 PathScan ELISA (Figure 3b).

Following this initial screening, the most effective candidate, VMP3, was selected for further evaluation. Its potential cytotoxic effects at higher concentrations were assessed using the MTT assay under serum‐containing conditions to monitor changes in metabolic activity.

Among the candidates, VMP3 consistently elicited the greatest proliferative response and a pronounced increase in VEGFR2 phosphorylation, supporting its selection for further characterization. Comprehensive screening results for all peptides are shown in Figure 3a–c. The EC_50_ value for VMP3 was calculated from proliferation data in the absence of VEGFR2 inhibitor (Figure 3d,e).

Beyond its proliferative effects, VMP3 significantly promoted EC migration, as demonstrated in both scratch wound and transwell chemotaxis assays. In the scratch assay, VMP3 accelerated wound closure across concentrations from 100 nM to 1 μM, with efficacy at 1 μM comparable to both VEGF (30 ng/mL) and the QK peptide (1 μM) (Figure 3f,g). Similarly, in transwell assays, VMP3 induced a robust, dose‐dependent increase in chemotactic migration, with activity that matched or exceeded the effects of VEGF and QK (Figure 3h,i). Together, these data establish that VMP3 effectively engages VEGFR2 and activates its downstream signaling cascade, acting through VEGFR2 to drive endothelial proliferation and migration processes fundamental to angiogenesis and tissue regeneration. Recombinant human VEGF was used as the VEGF ligand in all biological assays.

To further assess sequence specificity, an inactive peptide identified during the initial screening was used as a negative control. This peptide did not induce endothelial VEGFR2 phosphorylation under identical experimental conditions in VEGFR2 Pathscan ELISA, indicating that the biological activity observed with VMP3 is dependent on its specific sequence rather than the general effect of peptide exposure (Supplementary Figure S6).

The VMP3 concentration range used in this study (100 nM–1 μM) reflects standard practice for synthetic peptide agonists, which typically require higher molar concentrations than native protein ligands to achieve functional receptor activation. In contrast, VEGF ligand protein is a high‐affinity, dimeric growth factor that activates VEGFR2 at low mass‐based concentrations in the ng/mL range, whereas short peptides generally exhibit lower intrinsic affinity and lack bivalent receptor engagement. Accordingly, nanomolar‐to‐micromolar concentrations are commonly employed to assess the functional activity of VEGF‐mimetic peptides.

### 2.4 Biochemical and biophysical characterization of VMP3‐VEGFR2 interactions

**FIGURE 4:**
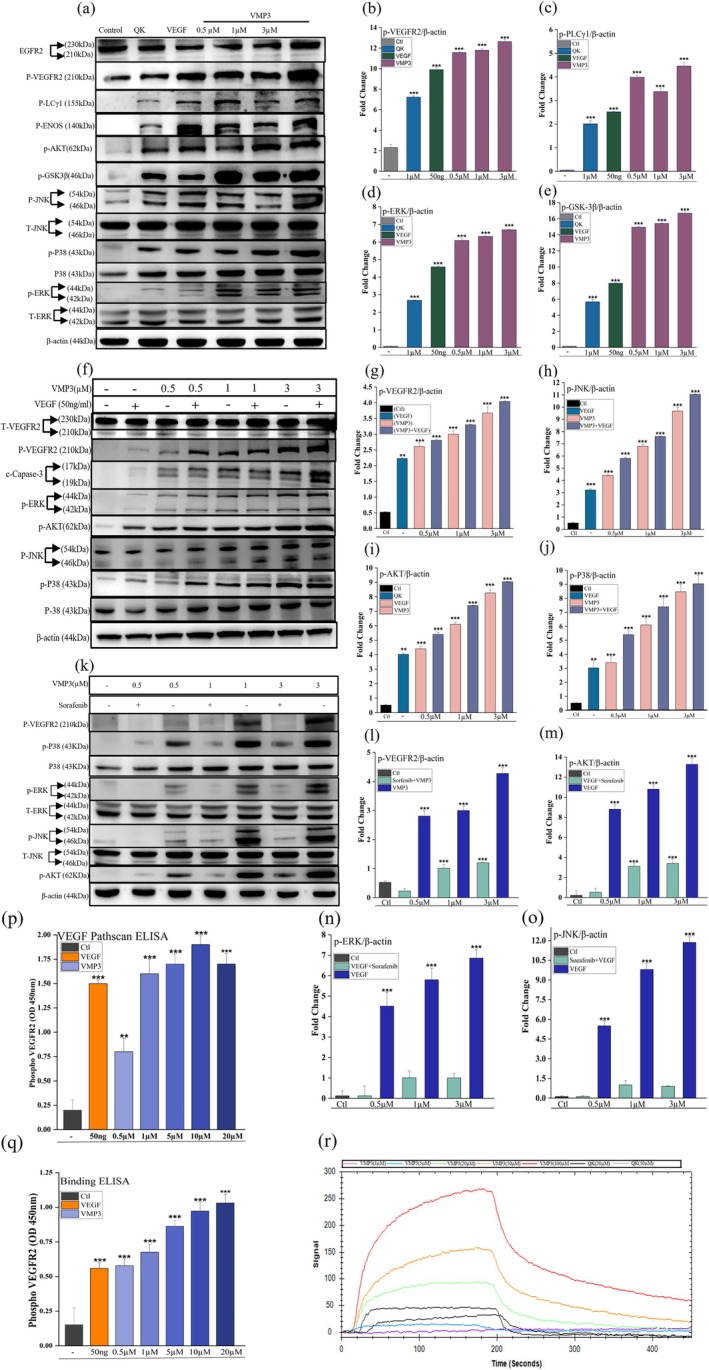
VMP3 activates VEGFR2 downstream signaling and binds specifically to the receptor. (a) Western blot showing dose‐dependent activation of VEGFR2 and downstream kinases (AKT, ERK1/2, and p38) upon VMP3 stimulation. (b)–(e) Densitometric quantification. (f) Western blot: VMP3 co‐treatment with VEGF. (g)–(j) Densitometric quantification. (k) Western blot: VMP3 with the VEGFR2 inhibitor sorafenib. (l)–(o) Densitometric quantification. (p) Binding ELISA at various VMP3 doses. (q) PathScan ELISA. (r) SPR analysis. All quantitative data (densitometric analyses, binding ELISA, and PathScan ELISA) are presented as mean ± SD from *n* = 3 independent experiments. Statistically significant differences were determined as *p<0.05, **p <0.01, *p <0.001 versus control groups using unpaired Student's *t*‐test and one‐way ANOVA (for western blot analyses) performed with GraphPad Prism v8 (La Jolla, CA, USA).

Western blot analysis revealed that VMP3 (0.5–3 μM) induced pronounced phosphorylation of VEGFR2 in HUVECs, accompanied by enhanced phosphorylation of multiple downstream effectors, including PLC*γ*1, eNOS, AKT, GSK3*β*, JNK, p38, and ERK1/2 (Figure 4a–e).

Co‐treatment with VEGF and VMP3 produced an additive increase in VEGFR2 phosphorylation, indicating potential synergy between the two agonists. Although VEGF and VMP3 both engage VEGFR2, synergistic signaling can arise even when ligands target the same receptor, as widely reported for receptor tyrosine kinases. VEGF ligand is a dimeric protein ligand that promotes canonical VEGFR2 dimerization and robust kinase activation. In contrast, VMP3 is a short peptide agonist that functions as a partial activator of VEGFR2 and does not require direct ligand‐mediated receptor crosslinking to elicit signaling. Accumulating evidence indicates that peptide ligands can modulate RTK activity by stabilizing signaling‐competent receptor conformations, enhancing receptor proximity or organization at the plasma membrane, and influencing the efficiency or duration of downstream signaling.

Consistent with this concept, VEGF‐mimetic peptides such as QK have been reported to enhance endothelial responses when used alone or in combination with VEGF, supporting functional cooperation rather than competitive receptor engagement (D'Andrea et al., 2005). In this context, VMP3 may act cooperatively with VEGF to potentiate VEGFR2 signaling, resulting in enhanced downstream pathway activation, as observed in Figure 4f–j. This response is therefore consistent with established models of functional cooperation within RTK signaling networks rather than competitive receptor engagement.

Western blot analysis demonstrated that VMP3 treatment induced phosphorylation of VEGFR2 and multiple downstream signaling molecules, including AKT, ERK1/2, PLC*γ*1, eNOS, and MAPK family members, indicating activation of canonical VEGFR2‐associated signaling pathways. Densitometric quantification of the remaining proteins is provided in Supplementary Figure S7.

The specificity of VMP3‐induced signaling was further validated using Sorafenib, a small‐molecule inhibitor of VEGFR tyrosine kinase activity. Treatment with Sorafenib markedly attenuated VEGFR2 phosphorylation and downstream pathway activation, including ERK, AKT, p38, and JNK signaling, while leaving total protein levels unchanged (Figure 4k–o), confirming that the observed responses are VEGFR2‐dependent and not due to nonspecific protein effects. While these data demonstrate broad activation of VEGFR2 downstream signaling, the relative contribution of individual pathways to the observed angiogenic responses was not dissected in the present study, and pathway‐specific inhibitor experiments will be important for future mechanistic resolution. While Sorafenib confirms that VMP3‐induced signaling is VEGFR2‐dependent, this approach does not selectively dissect the relative contribution of individual downstream pathways. Accordingly, pathway‐specific inhibition represents an important direction for future mechanistic studies.

These findings were validated by quantitative PathScan ELISA, which demonstrated a dose dependent increase in VEGFR2 phosphorylation following VMP3 treatment. Notably, at concentrations of 5 μM and above, VMP3 induced phosphorylation equaled or exceeded that observed with VEGF, with 10 μM identified as the optimal concentration for maximal receptor activation (Figure 4p).

Binding ELISA further confirmed that VMP3 directly and dose dependently interacts with VEGFR2, with 1 μM VMP3 producing an ELISA response comparable to that observed with 50 ng/mL VEGF ligand, and higher concentrations yielding increased signal intensity (Figure 4q). The use of QK as a positive control peptide reinforces VMP3's potential, as short peptides are generally associated with enhanced stability, lower immunogenicity, and more straightforward synthesis.

Surface plasmon resonance (SPR) analysis provided direct biophysical evidence of VMP3 and VEGFR2 interactions. Sequential injections of VMP3 produced concentration dependent sensograms characterized by rapid association and slow dissociation, indicative of strong and stable binding (Figure 4r). Kinetic analysis revealed that VMP3 binds VEGFR2 with nanomolar affinity (K_D = 1.60 nM), surpassing that of QK (Table 1). The high association rate, low dissociation rate, and substantial maximal binding capacity observed in SPR experiments support efficient and prolonged receptor engagement.

**TABLE 1:** SPR kinetic analysis of VMP3 binding to VEGFR2.

	Bmax (RU)	Ka (1/Ms)	Kd (1/s)	KD (M)	BI (RU)	Chi^2^ (RU^2^)
Mean	95.41	100,000.0	1.60e‐05	1.60e‐9	4.774	1.672
Std Dev	6.03	0	1.30e‐05	1.30e‐9	0.233	1.532

Although SPR with immobilized VEGFR2 does not fully replicate the dynamic dimerization that occurs in vivo, the robust and specific binding observed provides compelling evidence for VMP3's capacity to engage the VEGFR2 receptor effectively. The strength and stability of these interactions support the ability of VMP3 to promote receptor activation, potentially by stabilizing signaling‐competent receptor conformations or facilitating productive receptor proximity, rather than through direct ligand‐mediated receptor crosslinking. The precise structural and biophysical basis of VMP3‐mediated VEGFR2 activation will be addressed in future studies using complementary approaches such as fluorescence resonance energy transfer or co‐immunoprecipitation.

### 2.5 Ex vivo angiogenesis and VEGFR2 signaling analysis using the mouse aortic ring assay

**FIGURE 5:**
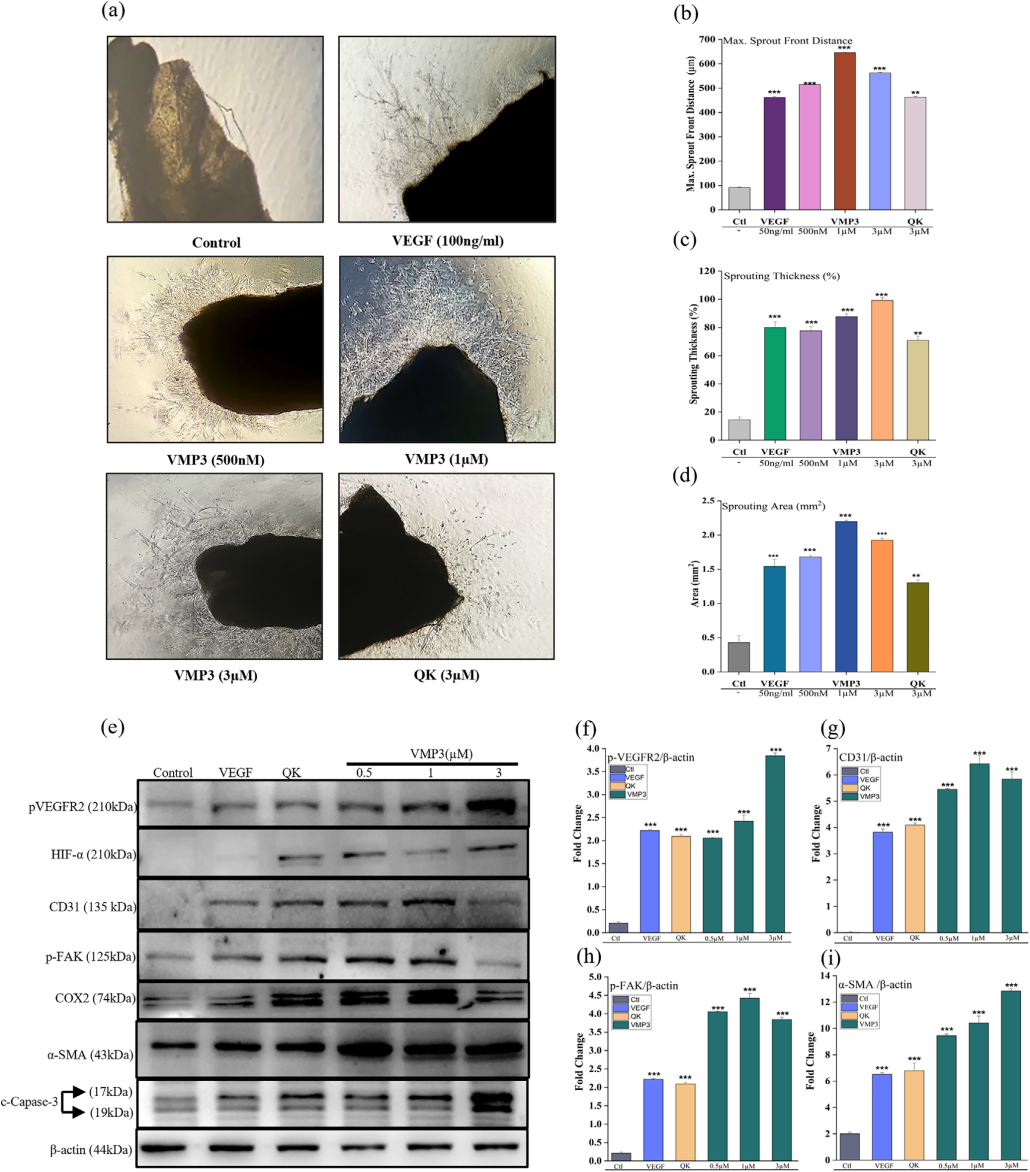
Ex vivo validation of VMP3‐induced angiogenesis and molecular marker expression. (a) Phase‐contrast images showing microvessel sprouting from mouse aortic rings treated with VEGF (100 ng/mL), VMP3 (500 nM or 100 nM), or QK peptide. (b)–(d) Quantification: Sprout front distance, thickness, and area. (e) Immunoblot analysis of angiogenic markers performed on the same mouse aortic ring samples and under the same treatment conditions as shown in panel (a). (f)–(i) Corresponding densitometric quantification normalized to *β*‐Actin. Data are presented as mean ± SD from *n* = 3 independent experiments. *p <0.05, **p <0.01, ***p <0.001 versus control, analyzed using unpaired Student's t‐test and one‐way ANOVA (for western blot analyses) with GraphPad Prism v8 (La Jolla, CA, USA).

The pro angiogenic potential of VMP3 was evaluated using a mouse aortic ring assay, an ex vivo model enabling direct observation and quantification of microvessel sprouting. Treatment with VMP3 (1 and 3 μM) significantly enhanced microvessel formation compared with the control (Figure 5a). Quantitative analysis (Figure 5b–d) demonstrated increases in maximum sprout front distance, sprout thickness, and total sprout area, confirming a strong angiogenic response.

At 1 μM, VMP3 induced high sprouting density, with predominantly short and highly branched microvessels. At 3 μM, microvessels were elongated, thicker, and morphologically mature, closely resembling VEGF treated positive controls. The angiogenic effect of VMP3 at 3 μM was comparable to VEGF, whereas QK induced a moderate response with less pronounced sprouting. Representative images of whole mouse aortic rings are shown in Figure S8. These results confirm that VMP3 robustly induces microvessel sprouting in an ex vivo model, consistent with its VEGFR2 activation profile.

To assess VEGFR2 pathway activation in microvessels formed in the mouse aortic ring assay, lysates from isolated sprouts were subjected to immunoblotting with antibodies targeting key signaling components. Analyses included phospho VEGFR2 (Tyr1175) and total VEGFR2 for receptor activation, and phospho AKT (Ser473) and total AKT for the PI3K/AKT pathway. Phospho ERK1/2 (Thr202/Tyr204) and total ERK1/2 were evaluated to monitor MAPK/ERK signaling, which is fundamental for endothelial proliferation and angiogenesis. *β*‐actin was used as a loading control to ensure equal protein input (Figure 5e). The expression levels of key proteins are graphically presented in Figure 5f–i.

Additional analyses of phospho JNK (Thr183/Tyr185) and phospho p38 (Thr180/Tyr182) provided insights into broader MAPK pathway activation and cellular stress responses. Enhanced chemiluminescence was used for band detection, and densitometric analysis was used to quantify relative protein expression. Collectively, these analyses elucidated the molecular mechanisms by which VMP3 modulates VEGFR2‐dependent angiogenic signaling in an ex vivo vascular sprouting model.

### 2.6 Assessment of in vivo angiogenesis via the mouse Matrigel plug assay

**FIGURE 6:**
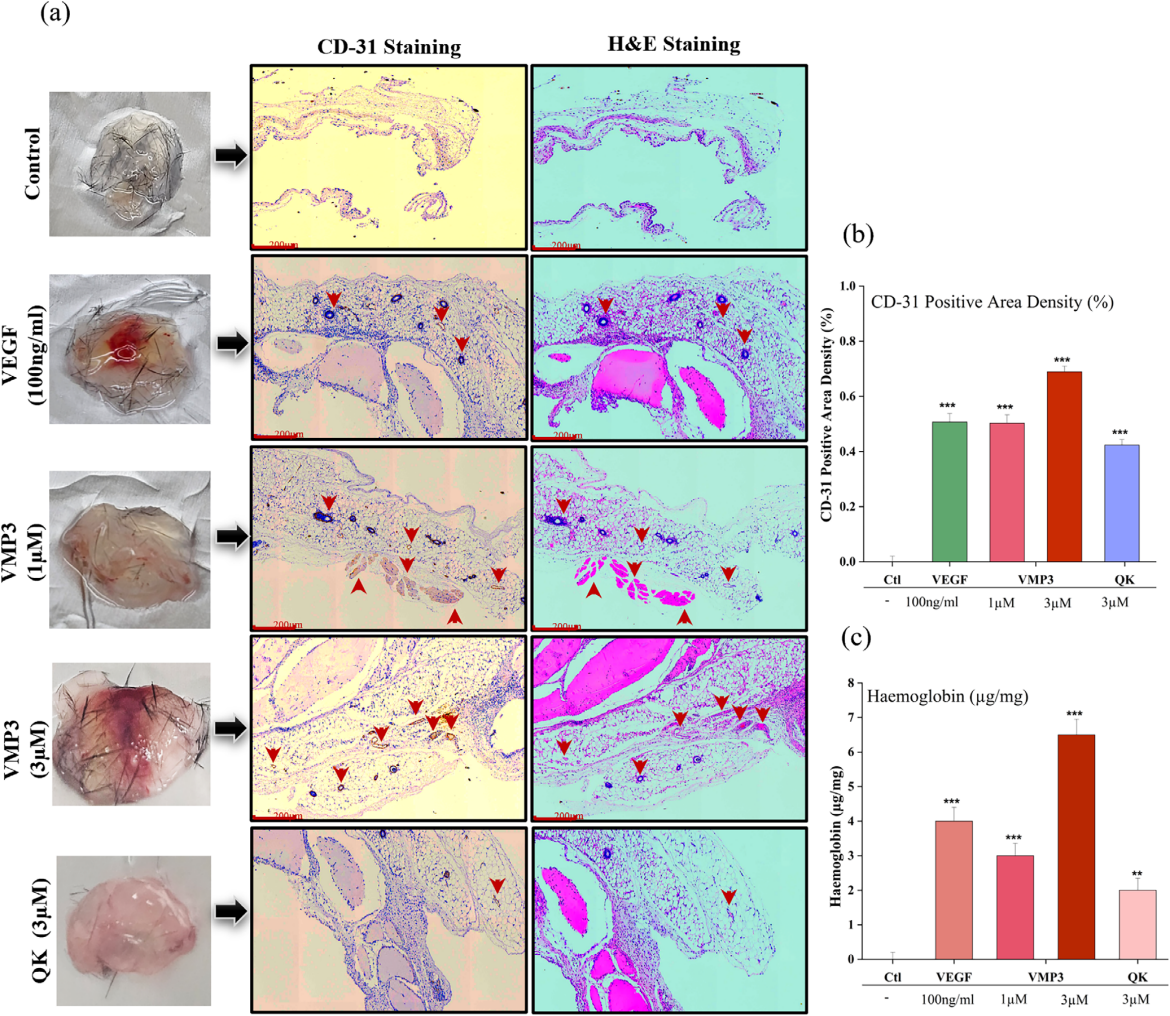
In vivo angiogenic potential of VMP3 assessed by a Matrigel plug assay. (a) Representative images of Matrigel plugs excised from mice after 14 days of subcutaneous implantation, showing gross morphology (left), CD31 immunostaining (middle), and hematoxylin–eosin (H&E) staining (right). (b) Quantification of CD31 positive area density (%), and (c) Quantification of hemoglobin content (μg/mg) in Matrigel plugs. Data are presented as mean ± SD from *n* = 3 independent experiments. *p <0.05, **p <0.01, ***p <0.001 versus untreated control, analyzed using unpaired Student's *t*‐test with GraphPad Prism v8 (La Jolla, CA, USA).

The angiogenic activity of VMP3 was evaluated using a Matrigel plug assay. Gross examination of excised plugs revealed enhanced vascularization in VMP3 treated groups (1 and 3 μM), whereas control plugs remained largely avascular (Figure 6a left panel). Plugs treated with VMP3 or VEGF exhibited a reddish hue, indicating blood vessel formation, in contrast to the pale and translucent appearance of the negative control.

Histological analysis confirmed the presence of newly formed vessels in VMP3 treated plugs, as indicated by red arrowheads (Figure 6a, right panel), while controls lacked evidence of vascularization. CD31 immunostaining further highlighted ECs lining the nascent vessels (Figure 6, mid panel) with increased CD31 expression reflecting higher vessel density and active angiogenesis (Yang et al., 1999). The angiogenic response was dose‐dependent, with 3 μM VMP3 inducing greater vascularization than 1 μM and comparable to VEGF, as supported by CD31 quantification (Figure 6b). Representative CD31‐stained images at a 60 μm scale, illustrating the distribution of CD31‐positive cells across all treatment groups, are presented in Figure S9.

Hemoglobin content analysis corroborated these findings, showing elevated levels in VMP3 treated plugs, indicative of functional perfused vessels (Figure 6c). Collectively, the gross morphological, histological, immunohistochemical, and quantitative data confirm that VMP3 effectively promotes in vivo angiogenesis, supporting its potential as a VEGF mimetic agent for vascular regeneration.

### 2.7 VMP3 promotes wound healing in diabetic mice

Type I diabetes is an autoimmune disorder characterized by the destruction of pancreatic *β*‐cells, leading to absolute insulin deficiency and persistent hyperglycemia (Ramalho et al., 2018). Impaired wound healing is a hallmark of type I diabetes, which contributes to chronic ulcers, delayed tissue repair, and secondary complications (Okonkwo & Dipietro, 2017).

**FIGURE 7:**
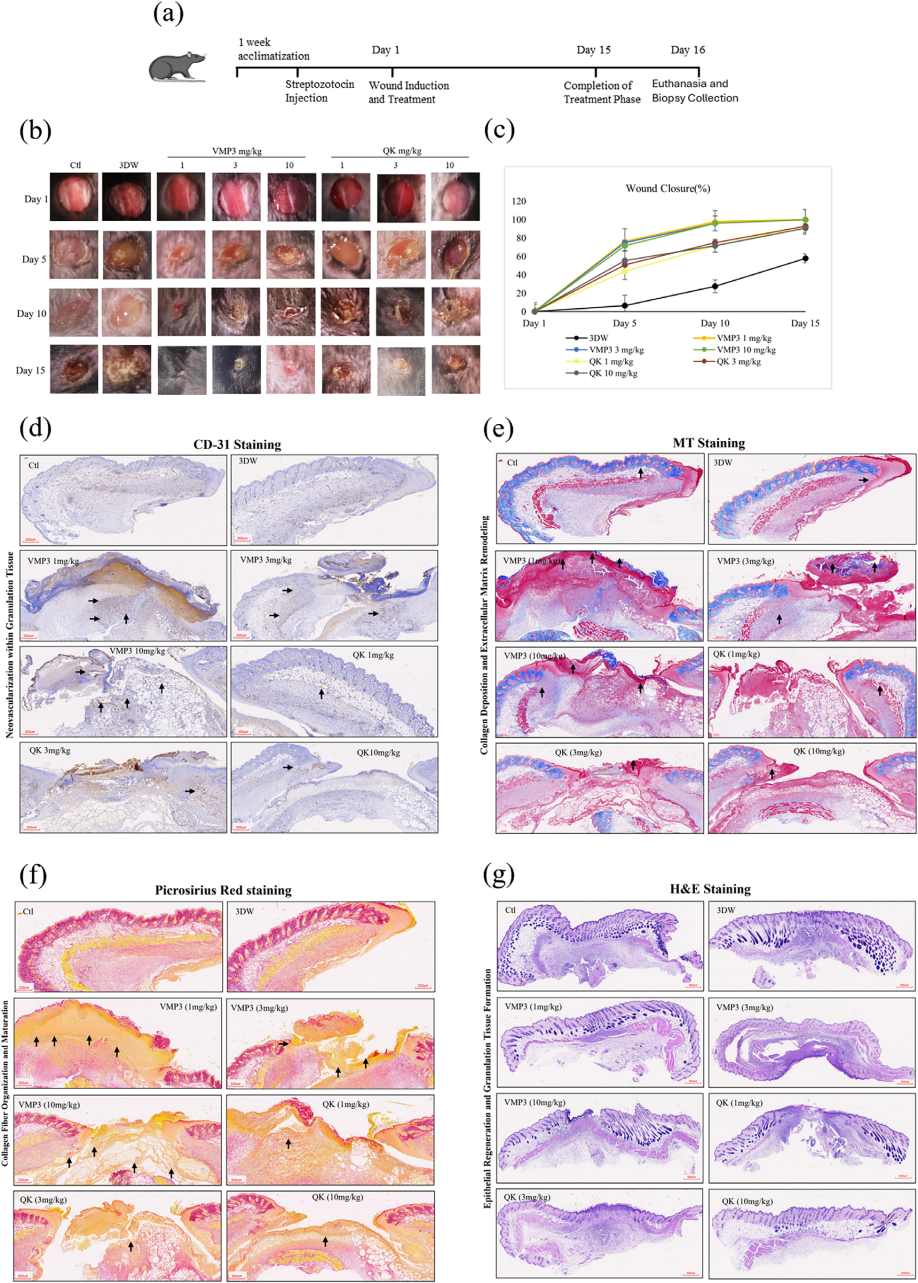
In vivo therapeutic evaluation of VMP3 in promoting wound healing in diabetic mice. (a) Schematic diagram illustrates the experimental design for diabetic wound induction and topical treatment with VMP3 in streptozotocin‐induced diabetic mice. (b) Representative wound images on Days 1, 5, 10, and 15 showing progressive wound closure across treatment groups. (c) Wound closure percentage profiles across treatment groups. Each treatment group consisted of five mice (*n* = 5). Data are presented as mean ± SD; *p* <0.05 indicates statistical significance (d) CD‐31 staining of mice wound tissues (day‐5) (e) MT staining of mice wound tissues (day‐5) (f) Picrosirius staining of mice wound tissues (day‐5) (g) Hematoxylin and Eosin (H&E) staining (Day 15) demonstrating overall tissue regeneration and wound closure (day‐15).

The therapeutic effect of VMP3 was examined in a streptozotocin (STZ) induced mouse model of type 1 diabetes. A schematic of the whole‐animal experimental procedure is presented in Figure 7a. Compared with the vehicle (3DW; distilled deionized water), disease, and positive control groups, VMP3 accelerated wound closure on Days 3, 5, and 7, with complete re‐epithelialization achieved by Day 15 at all doses as shown in Figure 7b. Histological analysis (H&E, Day 15) showed improved re‐epithelialization and higher keratinocyte and fibroblast proliferation in VMP3 treated wounds. Because angiogenesis is critical for repair, CD31 immunostaining was performed to evaluate neovascularization. VMP3 induced extensive capillary formation and tissue regeneration across all doses, exceeding that of QK, vehicle, and disease controls. CD31‐positive vessels formed dense, continuous networks in VMP3 treated wounds. Wound closure analysis on Day 15 showed near complete closure in all VMP3 treated groups, whereas residual open areas persisted in control wounds as depicted in Figure 7c.

Matrix remodeling was further supported by Masson's trichrome and Sirius Red staining, both revealing dose dependent collagen accumulation. Sirius Red indicated thicker, aligned collagen bundles, whereas Masson's trichrome confirmed enhanced matrix organization, collectively reflecting superior tissue repair and maturation following VMP3 treatment as demonstrated in Figure 7.

One of the major causes of delayed wound healing in diabetic conditions is the attenuated pro‐angiogenic phase of the repair process (Okonkwo & Dipietro, 2017). Previous studies have emphasized the critical role of VEGF in promoting angiogenesis and accelerating wound closure in diabetic models (Rai et al., 2022; Saaristo et al., 2006). In the present study, VMP3 treatment markedly enhanced neovascular formation within the wound tissue Figure 7d–g. The peptide promoted angiogenesis and tissue regeneration, both of which are essential components of effective wound healing (Okonkwo et al., 2020). Enhanced capillary density and organized vascular architecture observed in VMP3‐treated wounds indicate active endothelial proliferation and vessel maturation. Furthermore, histological evaluation revealed increased extracellular matrix accumulation and collagen fiber deposition, consistent with advanced tissue remodeling and structural recovery (Durr et al., 2025; Wang et al., 2025). The corresponding whole‐slide images of CD31, Masson's Trichrome, and Picrosirius Red staining are provided in Supplementary Figure S10. These findings demonstrate that VMP3 may exhibit VEGF‐mimetic properties, activating angiogenic signaling pathways and promoting matrix regeneration essential for wound repair in diabetic environments.

## 3 CONCLUSION, LIMITATIONS AND FUTURE DIRECTIONS

Future studies may further explore the effects of VMP3 in models of ischemic or chronic injury to better assess its translational relevance for vascular regeneration and wound repair. The identification of VMP3 as a partial VEGFR2 agonist offers insight into peptide‐mediated modulation of angiogenic signaling and provides a basis for the continued development and optimization of peptide‐based approaches for vascular regeneration.

Despite these encouraging observations, several translational considerations merit attention. As a peptide‐based agent, VMP3 may be subject to limitations related to in vivo stability, susceptibility to proteolytic degradation, and a relatively short pharmacokinetic half‐life. Although short peptides are generally associated with lower immunogenicity compared with full‐length proteins, additional studies will be necessary to comprehensively assess safety and immunogenic potential in relevant biological systems. While biophysical analysis such as SPR confirms direct binding of VMP3 to VEGFR2, these approaches do not directly assess receptor dimerization or binding cooperativity, and mechanistic interpretations related to these processes should therefore be regarded as hypothesis.

Future investigations may therefore focus on further delineating the structural and biophysical basis of VMP3‐mediated VEGFR2 activation, including targeted mutational analyses of residues such as Phe3 and Val6 to experimentally evaluate the computational predictions presented here. Moreover, assessment of peptide stability, biodistribution, and therapeutic performance in additional ischemic and chronic injury models would be informative. Collectively, these findings support continued investigation into peptide‐based modulation of angiogenic signaling as a potential strategy for vascular regeneration and wound repair.

## 4 MATERIALS AND METHODS

### 4.1 Data collection

Peptide design was based on the well‐established role of VEGF‐A in angiogenesis, where it binds to the KDR and Flt‐1 receptors on ECs, triggering key cellular processes such as proliferation, migration, and survival. Specifically, the 17–25 *α*‐helical region of VEGF‐A interacts with Flt‐1, a crucial step in the angiogenic signaling pathway. QK, a VEGF mimetic, mimics the 17–25 *α*‐helical region of VEGF‐A and binds to both VEGFR‐1 and VEGFR‐2 (D'Andrea et al., 2005). To identify potential VEGF agonistic peptides, a motif search was conducted using the Molecular Operating Environment (MOE) version 2020 (Chemical Computing Group, Montreal, Canada).

The search employed BLAST and pattern‐based sequence analysis, with default parameters. A 30% sequence identity threshold was applied, and only sequences below this threshold were retained to maximize sequence diversity and minimize redundancy. Although this threshold appears low for short peptides, it was intentionally selected to avoid trivial variants of known VEGF mimetics, as functional agonistic activity in short peptides is often governed by localized hotspot motifs rather than global sequence similarity.

Sequences from the Protein Data Bank (PDB) were selected for their meaningful variability, with a focus on *α*‐helical structures relevant to peptide design (Tsai et al., 2022). After removing redundant sequences, the final dataset included 81 peptides, ranging from 8 to 20 amino acids in length.

### 4.2 Training of recurrent neural network (RNN) and MD simulation

RNNs are designed to capture dependencies in sequential data (D'Andrea et al., 2005); however, traditional RNNs often struggle with long‐range dependencies due to vanishing or exploding gradients (He et al., 2016). LSTM networks, an improved variant of RNNs, overcome these limitations through gating mechanisms: input, forget, and output gates which regulate the flow of information and enable the capture of long‐term dependencies in peptide sequences (Staudemeyer & Morris, 2019).

In this study, the LSTM model was implemented using TensorFlow and Keras. Peptide sequences were processed at the amino‐acid (character) level and encoded using a trainable embedding layer, which mapped each residue into a 50‐dimensional dense vector space. These embeddings were learned jointly with model parameters during training, enabling contextual sequence representation beyond fixed one‐hot or physicochemical encodings.

The network architecture consisted of a single LSTM layer with 64 memory units followed by a dense output layer with SoftMax activation to predict the probability distribution of the subsequent amino acid in a sequence. Model training was performed using the Adam optimizer and sparse categorical cross‐entropy loss, with the objective of next amino acid prediction. The model was trained for 200 epochs using a batch size of 32, with convergence observed within the first ~50 epochs as presented in supplementary Table S2–S4.

Peptide design was performed using an AI‐assisted workflow integrating LSTM‐based sequence generation with physicochemical filtering and structure‐guided refinement. Candidate sequences generated by the LSTM model were filtered based on predefined physicochemical criteria, including peptide length (≥10 amino acids), net charge (−2 to +2 at physiological pH), and GRAVY score (−1.0 to +1.0). Filtered sequences were evaluated for structural compatibility with VEGF‐mimetic design principles, guided by conserved features of the *α*‐helical 17–25 region of VEGF‐A and by known VEGF‐mimetic peptides such as QK. Rational point mutations were introduced at selected positions to adjust charge distribution, hydrophobic balance, or predicted helical stability. Sequences failing to meet these criteria were excluded prior to molecular docking analysis. A final subset of six peptides (VMP1–VMP6) was selected for synthesis and experimental evaluation. Peptide‐specific physicochemical properties, in silico selection rationale, and experimental outcomes are summarized in Supplementary Table S1.

Given the relatively small dataset size (81 peptides), the LSTM model was not intended to predict biological activity directly, but rather to learn sequence‐level patterns within a constrained VEGF‐mimetic peptide space. To assess generalization and mitigate overfitting, the dataset was divided into training and hold‐out test subsets, and model performance was monitored on both sets.

Following training, the model generated novel peptide sequences by iteratively predicting successive amino acids from an initial seed. Each sequence was systematically evaluated for key physicochemical properties molecular weight, hydrophobicity (GRAVY score), net charge, and isoelectric point using the Protein Analysis module in Biopython.

Several promising sequences were identified, and one top‐ranked peptide was further optimized using MOE software. Rational point mutations were introduced based on conserved motifs and structural features of known VEGF mimetic peptides, representing an AI‐assisted, human‐guided optimization step, yielding the optimized peptide VMP3, which retained favorable physicochemical properties and closely resembled the target biological motif.

To facilitate prioritization for molecular docking, all generated sequences were compared to a known VEGF mimetic peptide (“KLTWQELYQLKYKGI”) using Levenshtein distance as a structural similarity metric. Peptides satisfying predefined physicochemical thresholds length ≥10 residues, net charge −2 to +2, and GRAVY score −1.0 to +1.0 were ranked by normalized Levenshtein similarity, and the top candidates were selected for downstream molecular modeling and simulation.

**FIGURE 8:**
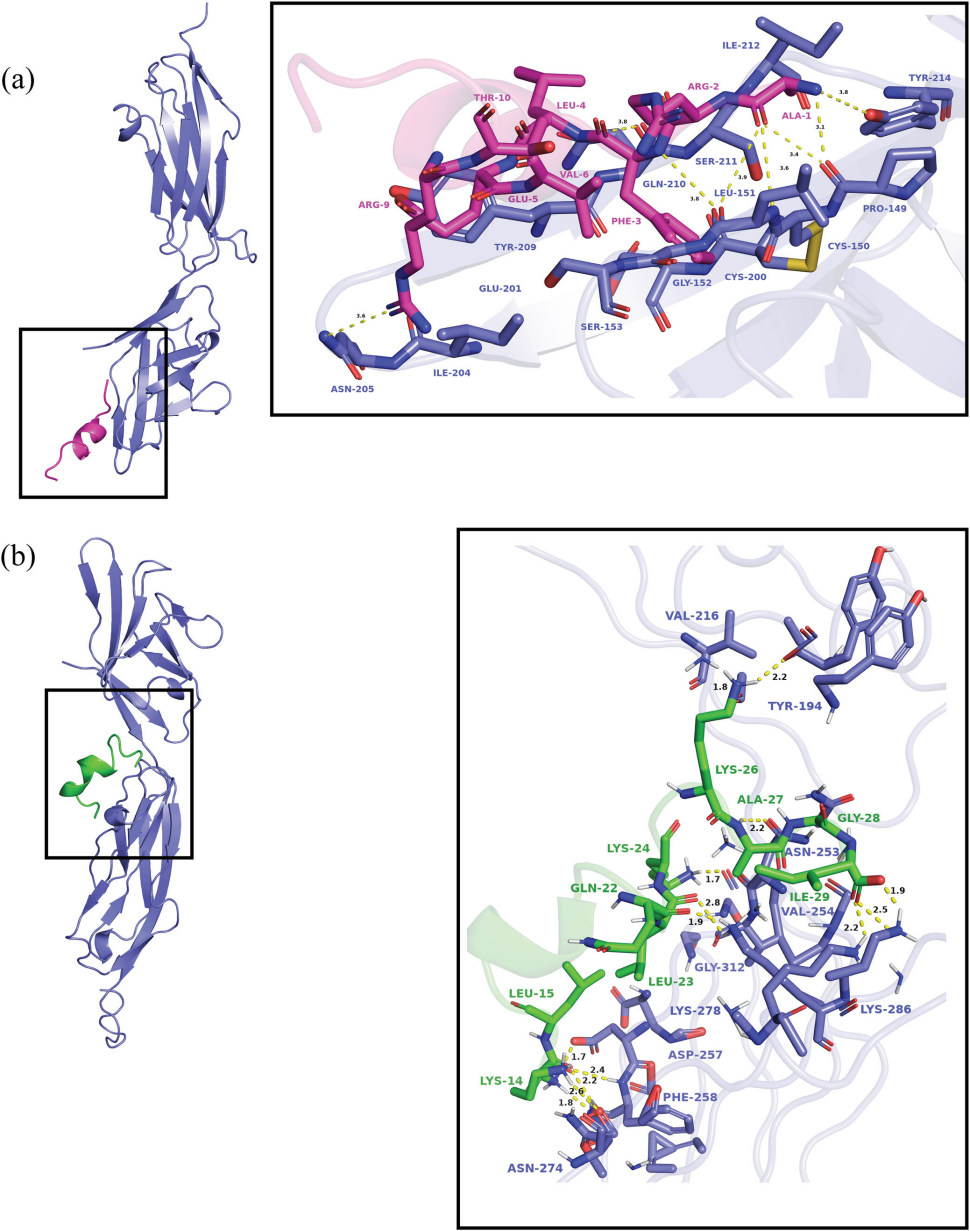
Protein‐peptide interactions with VEGF‐R2. (a) Interaction of the VMP3 peptide (magenta) with the VEGF‐R2 receptor (blue), highlighting key interacting residues and hydrogen bonds (yellow dashed lines), and (b) interaction of the QK peptide (green) with the VEGF‐R2 receptor (blue), showing key residue interactions and hydrogen bond distances.

MD simulations were performed to investigate protein‐peptide interactions at atomic resolution (Martino et al., 2011). The conformations of VMP3 and QK bound to VEGFR‐2 (PDB ID: 3V2A) served as model structures (Figure 8a,b), with peptide sequences and molecular weights listed in Table 2. Complexes were solvated in a cubic box with a minimum 0.5 Å (0.05 nm) buffer from the box edges. Energy minimization was performed using the steepest descent algorithm until the target force fell below 1000 kJ·mol^−1^·nm^−1^. Equilibration comprised a 1000 ps NVT ensemble with V rescale thermostat, followed by a 1000 ps NPT ensemble using Parrinello–Rahman pressure coupling. Both phases maintained a reference temperature of 300 K with a coupling time constant of 0.1 ps; NPT equilibration employed a reference pressure of 1 bar and a compressibility of 4.5 × 10^−5^ bar^−1^. The source code and LSTM training dataset are available at GitHub (https://github.com/Rameezlatif/LSTM_VEGF_Final).

**TABLE 2:** Peptide sequences of VMP3 and QK.

Sr. No.	Name	Sequence	Molecular weight (Da)
1	VMP3	ARFLEVWQRTYCKA	1810
2	QK	KLTWQELYQLKYKGI	1911

The production MD simulations were performed at 300 K using a 2‐fs time step. The CHARMM36 (Chemistry at Harvard macromolecular mechanics) force field (Huang & Mackerell, 2013) and CHARMM modified version of TIP3P water model were employed (Boonstra et al., 2016). Electrostatic interactions were calculated using the particle mesh Ewald method with a short‐range cutoff of 1.0 nm. Constraints on hydrogen‐containing bonds were applied using the LINCS algorithm, with an order of four and a single iteration for accuracy. The Verlet cutoff scheme was used for neighbor searching, with a grid‐based search performed every 20 fs. The initial comparative dynamics simulation of QK and VMP3 with VEGFR2 lasted for 100 ns; later, extended simulations (300 ns triplicates) were performed in case of VMP3 to gain its binding insights.

### 4.3 Experimental reagents and cell handling

A detailed list of chemicals and reagents utilized in this study is available in the Supplementary Data. Analytical profiles, including HPLC and mass spectrometry (MS) graphs, are presented in Figure S11a,b.

#### 4.3.1 Cell culture

HUVECs (ATCC, USA) were cultured in VEGF‐free vascular basal medium with an endothelial growth kit (containing FGF). Cellular effects were attributed to experimental treatments, with VMP3 activity compared to untreated controls. Medium was changed after every 2 days. Cells at ~80% confluence were passaged 1:3 using 0.25% trypsin (EDTA free). Cultures were maintained at 37°C in 5% CO_2_.

### 4.4 Cellular functional assays

#### 4.4.1 Cell viability and cell proliferation

HUVECs were seeded at 0.5 × 10^4^ cells/well in 96 well plates (BD Biosciences, San Jose, CA, USA) and incubated overnight for attachment. The next day, the cells were exposed to various concentrations of VMP3, VEGF, QK, and the negative control (untreated), for 24 h. Cell viability was determined using the colorimetric 1‐(4,5‐dimethylthiazol‐2‐yl)‐3,5‐diphenylformazan [MTT] assay (Sigma Aldrich) (Mosmann, 1983). Following treatment, the medium was removed, and the intracellular formazan crystals were solubilized in DMSO, with absorbance measured at 570 nm using a microplate reader. To evaluate the effects of the peptide on cell proliferation, cells were serum‐starved for 6 h, treated with the peptide in serum‐free medium for 24 h, and assessed for viability using the Trypan Blue exclusion assay.

#### 4.4.2 Scratch wound healing

HUVECs were seeded at 2 × 10^5^ cells/well in 24 well plates and cultured to 70%–80% confluence. Cell proliferation was inhibited using mitomycin (10 μg/mL) for 3 h, followed by washing with 1× PBS. A uniform scratch was created using a sterile 200 μL pipette tip (Martinotti & Ranzato, 2020) and debris was removed by washing twice with PBS. Fresh serum free medium was added, and cells were treated with varying concentrations of VMP3, VEGF, QK, and untreated cells (negative control). Scratch areas were imaged at 0, 24, and 48 h using an inverted microscope, and cell migration was quantified using Image J by comparing initial and final scratch widths. The percentage closure of the scratch wound was calculated using the following equation:

(1) $$\text{Wound closure}\;\left(\%\right)=\frac{\mathrm{Ao}-\mathrm{At}}{\mathrm{Ao}}x\;100$$

Here, *A*_0_ is the initial wound area at 0 h, and *A*_*t*_ is the scratch area at 24 h. The experiment was performed in triplicate to ensure statistical accuracy.

#### 4.4.3 Chemotactic migration assessment using the transwell

HUVEC migration in response to VMP3 was evaluated using 24‐well transwell chambers with 8 μm pore, polycarbonate inserts (Corning, NY, USA) (Justus et al., 2023). Serum‐starved HUVECs were seeded in the upper chamber at 1 × 10^5^ cells/200 μL in serum free medium, while the lower chamber contained medium with varying concentrations of VMP3, VEGF, QK, and negative control (medium only). After 24 h at 37°C, non‐migrated cells were removed, and migrated cells on the lower membrane were fixed with 4% paraformaldehyde (15 min), stained with 0.1% crystal violet (30 min), washed, and air dried. Migrated cells were counted under a light microscope (200×) in five random fields per membrane.

### 4.5 Evaluation of VEGFR2 phosphorylation and downstream signaling

#### 4.5.1 Western blot analysis

HUVECs (5 × 10^5^ per dish) were seeded in 6 cm dishes, cultured for 24 h, and serum starved for 6 h. Cells were treated for 30 min with VMP3, VEGF, QK, and untreated cells (negative control). Parallel treatments included VEGF and the VEGFR inhibitor Sorafenib (5 μM) to assess signaling modulation (42). Cells were lysed using NE‐PER™ Nuclear and Cytoplasmic Extraction Reagents (Thermo Fisher) with protease and phosphatase inhibitors. Protein concentrations were determined by BCA assay. Samples (50 μg for VEGFR2, 30 μg for other markers) were resolved by 10% SDS PAGE and transferred to nitrocellulose membranes. Membranes were blocked in 5% nonfat milk, incubated overnight at 4°C with primary antibodies (1:1000), and then with HRP conjugated secondary antibodies (1:1000, 2 h, room temperature). Detection used SuperSignal West Pico Plus substrate (Thermo Scientific) and imaging with a Bio Rad ChemiDoc Touch system. *β*‐actin was used as a loading control. All experiments were performed in triplicate.

#### 4.5.2 VEGFR2 path scan enzyme linked immunosorbent assay (ELISA)

Phosphorylated VEGFR2 (Tyr1175) levels were quantified using the PathScan® ELISA Kit (CST, 7335CA) per the manufacturer's instructions. HUVECs were serum starved, treated with VMP3, VEGF, QK, lysed, and total protein measured by BCA assay. Equal protein amounts were loaded to microplate wells pre‐coated with a VEGFR2 capture antibody. Following incubation, a phospho‐specific anti‐VEGFR2 (Tyr1175) rabbit detection antibody was added, followed by an HRP‐conjugated secondary antibody supplied with the kit. Signal development was measured at 450 nm using a BioTek Synergy H1 microplate reader. All assays were performed in triplicate and independently repeated three times.

### 4.6 Evaluation of VMP3 binding affinity and kinetics

#### 4.6.1 Binding ELISA

An ELISA was employed to examine the binding affinity of VMP3 for recombinant VEGFR2. Plates coated with VEGFR2 (200 ng/well) were blocked and incubated with graded concentrations of VMP3 (1–50 μM) for 2 h. After removal of unbound peptide, anti‐VEGF primary and HRP‐conjugated secondary antibodies were applied. Color development was achieved using TMB substrate, and absorbance was measured at 450 nm. Assays were conducted in triplicate, with binding responses compared to those of QK, VEGF, and a negative control.

#### 4.6.2 SPR analysis

Binding affinity studies were performed using a Reichert SR7500DC SPR system. Recombinant human VEGFR2 was immobilized on an iClueBio HC1000 sensor chip (HCCH101KX) through EDC/NHS‐mediated amine coupling, followed by blocking with 1 M ethanolamine (pH 8.5). Interaction kinetics between the VEGFR2 and VMP3 were assessed in 1× PBS at a flow rate of 50 μL/min, with each binding cycle lasting for about 500 s. Reference subtracted response signals were analyzed with a 1:1 Langmuir binding model to calculate association (*k*ₐ), dissociation (k_d), and equilibrium dissociation (K_D) constants. Kinetic parameters were determined by nonlinear regression with 95% confidence intervals for comparative binding affinity assessment.

### 4.7 Mice

Female C57BL/6J mice (7 weeks old) were obtained from Orient Bio Inc. (Seongnam, South Korea) and housed at the Animal Experiment Center of Ajou University under standard conditions with ad libitum access to food and water. All procedures were approved by the Institutional Animal Care and Use Committee of Ajou University (IACUC 2024‐0052) and conducted following established ethical guidelines for animal research.

### 4.8 Ex vivo evaluation of angiogenic potential

#### 4.8.1 Mouse aortic ring assay

Matrigel, which mimics the natural extracellular matrix, is commonly used to study angiogenesis in vitro and in vivo (Kastana et al., 2019). The angiogenic activity of VMP3 was assessed using an ex vivo mouse aortic ring assay (Masson et al., 2002). Aortas were harvested from 7‐week‐old female C57BL/6J mice in accordance with institutional ethical guidelines, cleared of connective tissue, and sectioned into ~1‐mm rings. Each ring was embedded in Matrigel in 24 well plates, precoated for 30 min, and overlaid with serum free medium containing vehicle control, 3DW, VMP3 (1 or 3 μM), QK (3 μM), or VEGF (100 ng/mL). Plates were incubated at 37°C in 5% CO_2_. Rings were centrally positioned and sealed with an additional Matrigel layer. VEGF free vascular basal medium was added and replaced every 3 days. After 10 days, sprouting microvessels were imaged at 4× magnification, and vessel formation and morphology were quantitatively analyzed. Each condition included at least three aortic rings.

#### 4.8.2 Analysis and quantification of mouse aortic rings

Vessel sprouting parameters, including maximum sprout front distance, sprout length, and total sprout area were quantified using the Wimasis Image Analysis platform (Wim Sprout Module, Córdoba, Spain) (https://www.wimasis.com/, n.d.). Microscopic images (4× magnification) were processed by automated segmentation to delineate sprout boundaries, with all groups analyzed in parallel to minimize batch effects (Figures S12 and S13). Maximum sprout front distance represented the furthest extension from the ring; sprout length, the longest vessel; and total area, the sum of microvessel regions. Quantitative data were exported from Wimasis and cross validated by manual tracing on three randomly selected images per group. All measurements were performed in a blinded fashion and are reported as mean ± SD from three independent experiments, each including at least three rings per group. This approach ensured objective, high throughput analysis and minimized observer bias.

#### 4.8.3 Analysis of VEGFR2 signaling pathway in aortic ring microvessels

For analysis of VEGFR2 signaling in aortic ring derived microvessels, proteins were extracted from sprouting tissues using RIPA buffer (Thermofisher) with protease and phosphatase inhibitors. Protein concentrations were subsequently determined using a BCA assay. Equal amounts of protein were separated by SDS PAGE and transferred to PVDF membranes, which were blocked with 5% BSA in 1× PBST. Membranes were incubated overnight at 4°C with primary antibodies against p‐VEGFR2, HIF‐*α*, CD31, p‐FAK, COX2, *α*‐SMA, cleaved caspase‐3, and *β*‐actin, followed by 2 h incubation with HRP‐conjugated secondary antibodies at room temperature. Protein bands were visualized using enhanced chemiluminescence (ECL), and band intensities were quantified using Image J software.

### 4.9 In vivo analysis of angiogenesis through the mouse Matrigel plug assay

The angiogenic activity of VMP3 was examined using the Matrigel plug assay approved by the IACUC of Ajou University Medical Center. Female C57BL/6 mice (6–8 weeks) were acclimated for 1 week and randomly assigned (*n* = 5). Matrigel (800 μL; Corning) containing VMP3 (1 or 3 μM), VEGF (100 ng/mL), QK peptide (3 μM), or vehicle (3DW) was injected subcutaneously into the flank. Plugs solidified in situ, and mice were monitored daily.

After 8 days, mice were euthanized by CO_2_ asphyxiation and plugs were collected, photographed, rinsed in 1× PBS, and analyzed for hemoglobin content using Drabkin's reagent (Sigma‐Aldrich) as a marker of neovascularization. Samples were fixed in 4% paraformaldehyde, paraffin‐embedded, sectioned (4 μm), and stained with H&E and anti‐CD31. Digital image analysis (GS Tech Korea Co., Ltd.) quantified CD31‐positive vessel number, density, diameter, and branching from 20× fields (Olympus IX73). H&E showed tissue structure, and CD31 confirmed endothelial vessel formation and VMP3‐induced angiogenesis.

### 4.10 Diabetes induction, wound model establishment, and peptide treatment

Experimental diabetes was established by intraperitoneal injection of freshly dissolved streptozotocin at 150 mg/kg. Blood glucose was assessed from tail‐tip blood samples on three consecutive occasions, and animals maintaining fasting glucose levels above 250 mg/dL were classified as diabetic and retained for subsequent experiments.

Diabetic C57BL/6J mice were randomly distributed into eight groups (*n* = 5) comprising a vehicle control (3DW), VMP3 treatment at 1, 3, and 10 mg/kg, and QK peptide at equivalent concentrations as a positive control. Following anesthesia, the dorsal surface was shaved, disinfected, and a circular full‐thickness excision (6 mm diameter) was made to generate the wound model. The designated peptide formulation or vehicle control was applied topically, and the wound was sealed with a sterile Tegaderm dressing. Digital images were obtained on Days 0, 5, 10, 14, and 15 to evaluate wound closure dynamics. On Day 16, animals were euthanized by CO_2_ inhalation, and skin specimens encompassing the wound margins were collected for histological and molecular analyses.

### 4.11 Statistical analysis

Statistical analyses were conducted using GraphPad Prism software (version 7.00; GraphPad Software, La Jolla, CA, USA). Data are presented as the mean ± standard deviation (SD) obtained from at least three independent experiments; each performed in three to five replicates. Statistical differences between the control and treatment groups were evaluated using an unpaired Student's *t*‐test and one‐way ANOVA with GraphPad Prism v8 (La Jolla, CA, USA).

## AUTHOR CONTRIBUTIONS

**Farzana Yasmeen:** Conceptualization; methodology; investigation; visualization; writing – original draft; writing – review and editing; formal analysis. **Rajath Ramachandran:** Validation; formal analysis; software; data curation; visualization. **Rameez Hassan Pirzada:** Software; formal analysis; visualization; conceptualization. **Bogeum Choi:** Formal analysis. **Hana Seo:** Formal analysis. **Wook Kim:** Conceptualization; validation. **Moon Suk Kim:** Conceptualization; validation. **Sangdun Choi:** Conceptualization; supervision; resources; writing – review and editing; validation.

## CONFLICT OF INTEREST STATEMENT

The authors declare that they have no known competing financial interests or personal relationships that could have appeared to influence the work reported in this paper.

## Supporting information


**Figure S1:** Molecular dynamics (MD) simulations of the VEGFR2–VMP3 complex.
**Figure S2**: Comparison of structural RMSD of VMP3 docked structure and final molecular dynamics structure.
**Figure S3**: Comparison of structural RMSD of VMP3 crystal structure and final molecular dynamics structure.
**Figure S4**: Comparison of structural RMSD of VMP3 bound VEGF‐R2 and apo form structure at 300 ns.
**Figure S5**: RMSD of VEGF‐R2 during molecular dynamics simulation.
**Figure S6:** PathScan ELISA analysis of VEGFR2 phosphorylation in response to VMP3 and a negative control peptide.
**Figure S7:** Densitometric Analysis of Western Blot Results.
**Figure S8:** Mouse Aortic Ring Assay (Whole section).
**Figure S9:** Background noise deletion and sprout vessels boundaries detection.
**Figure S10**: Sprout vessels area detection.
**Figure S11:** Distribution of CD31‐positive cells across different treatment groups, at scale 60 μm.
**Figure S12:** Whole‐slide images of wound tissues section.
**Figure S13**: HPLC and MS graphs of VMP3 synthesis.
**Table S1:** Physicochemical and experimental characterization of AI‐assisted VEGF‐mimetic peptides.
**Table S2**: Molecular dynamics parameters implemented for MD production.
**Table S3:** Binding free energy criteria.
**Table S4**: Site‐directed mutagenesis of energetically favorable residues at VMP3 binding site.

## Data Availability

All data that support the findings of this study are available within the supplementary materials accompanying this article.

## References

[pro70529-bib-0001] Ahmad B , Achek A , Farooq M , Choi S . Accelerated NLRP3 inflammasome‐inhibitory peptide design using a recurrent neural network model and molecular dynamics simulations. Comput Struct Biotechnol J. 2023;21:4825–4835. 10.1016/j.csbj.2023.09.038 37854633 PMC10579963

[pro70529-bib-0002] Boonstra S , Onck PR , van der Giessen E . CHARMM TIP3P water model suppresses peptide folding by solvating the unfolded state. J Phys Chem B. 2016;120:3692–3698. 27031562 10.1021/acs.jpcb.6b01316

[pro70529-bib-0003] Brozzo MS , Bjelić S , Kisko K , Schleier T , Leppänen VM , Alitalo K , et al. Thermodynamic and structural description of allosterically regulated VEGFR‐2 dimerization. Blood. 2012;119:1781–1788. 22207738 10.1182/blood-2011-11-390922

[pro70529-bib-0004] Carmeliet P , Jain RK . Molecular mechanisms and clinical applications of angiogenesis. Nature. 2011;473:298–307. Available from: https://www.nature.com/articles/nature10144 21593862 10.1038/nature10144 PMC4049445

[pro70529-bib-0005] Carmeliet P , Jain RK . Angiogenesis in cancer and other diseases. Nature. 2000;407:249–257. Available from: www.nature.com 11001068 10.1038/35025220

[pro70529-bib-0006] Cébe‐Suarez S , Zehnder‐Fjällman A , Ballmer‐Hofer K . The role of VEGF receptors in angiogenesis; complex partnerships. Cell Mol Life Sci. 2006;63:601. 10.1007/s00018-005-5426-3 16465447 PMC2773843

[pro70529-bib-0007] Claesson‐Welsh L , Welsh M . VEGFA and tumour angiogenesis. J Intern Med. 2013;273:114–127. 23216836 10.1111/joim.12019

[pro70529-bib-0008] Crawford T , Alfaro D III , Kerrison J , Jablon E . Diabetic retinopathy and angiogenesis. Curr Diabetes Rev. 2009;5:8–13. 19199892 10.2174/157339909787314149

[pro70529-bib-0009] D'Andrea LD , de Rosa L , Vigliotti C , Cataldi M . VEGF mimic peptides: potential applications in central nervous system therapeutics. New Horizons Transl Med. 2017;3:233–251. 10.1016/j.nhtm.2016.12.002

[pro70529-bib-0010] D'Andrea LD , Iaccarino G , Fattorusso R , Sorriento D , Carannante C , Capasso D , et al. Targeting angiogenesis: structural characterization and biological properties of a de novo engineered VEGF mimicking peptide. Proc Natl Acad Sci USA. 2005;102:14215–14220. 16186493 10.1073/pnas.0505047102 PMC1242306

[pro70529-bib-0012] de Rosa L , Finetti F , Diana D , di Stasi R , Auriemma S , Romanelli A , et al. Miniaturizing VEGF: peptides mimicking the discontinuous VEGF receptor‐binding site modulate the angiogenic response. Sci Rep. 2016;6:1–13. 27498819 10.1038/srep31295 PMC4976335

[pro70529-bib-0013] Deveza L , Choi J , Yang F . Therapeutic angiogenesis for treating cardiovascular diseases. Theranostics. 2012;2:801–814. 22916079 10.7150/thno.4419 PMC3425124

[pro70529-bib-0014] di Stasi R , de Rosa L , D'Andrea LD . Structure‐based design of peptides targeting VEGF/VEGFRs. Pharmaceuticals. 2023;16:851. 37375798 10.3390/ph16060851 PMC10302803

[pro70529-bib-0015] Durr HA , Abri S , Salinas SD , Adkins‐Travis K , Amini R , Shriver LP , et al. Extracellular matrix repair and organization of chronic infected diabetic wounds treated with methacrylated chitosan‐based hydrogels. Acta Biomater. 2025;199:166–177. 10.1016/j.actbio.2025.04.062 40318743 PMC12983406

[pro70529-bib-0016] Fallah A , Sadeghinia A , Kahroba H , Samadi A , Heidari HR , Bradaran B , et al. Therapeutic targeting of angiogenesis molecular pathways in angiogenesis‐dependent diseases. Biomed Pharmacother. 2019;110:775–785. 10.1016/j.biopha.2018.12.022 30554116

[pro70529-bib-0017] Ferrara N , Adamis AP . Ten years of anti‐vascular endothelial growth factor therapy. Nat Rev Drug Discov. 2016;15:385–403. 26775688 10.1038/nrd.2015.17

[pro70529-bib-0018] Finetti F , Basile A , Capasso D , Di Gaetano S , di Stasi R , Pascale M , et al. Functional and pharmacological characterization of a VEGF mimetic peptide on reparative angiogenesis. Biochem Pharmacol. 2012;84:303–311. 10.1016/j.bcp.2012.04.011 22554565

[pro70529-bib-0019] Fujii H , Sun Z , Li SH , Wu J , Fazel S , Weisel RD , et al. Ultrasound‐targeted gene delivery induces angiogenesis after a myocardial infarction in mice. JACC Cardiovasc Imaging. 2009;2:869–879. 10.1016/j.jcmg.2009.04.008 19608138

[pro70529-bib-0020] Galiano RD , Tepper OM , Pelo CR , Bhatt KA , Callaghan M , Bastidas N , et al. Topical vascular endothelial growth factor accelerates diabetic wound healing through increased angiogenesis and by mobilizing and recruiting bone marrow‐derived cells. Am J Pathol. 2004;164:1935–1947. 10.1016/S0002-9440(10)63754-6 15161630 PMC1615774

[pro70529-bib-0021] Geist C , Useini A , Kazimir A , Kümpfel R , Meiler J . Computer‐guided design of Z domain peptides with improved inhibition of VEGF. 2024;1:1–15.

[pro70529-bib-0022] Gers FA , Schmidhuber J , Cummins F . Learning to forget: continual prediction with LSTM. Neural Comput. 2000;12:2451–2471. 10.1162/089976600300015015 11032042

[pro70529-bib-0023] Gille H , Kowalski J , Li B , LeCouter J , Moffat B , Zioncheck TF , et al. Analysis of biological effects and signaling properties of Flt‐1 (VEGFR‐1) and KDR (VEGFR‐2): a reassessment using novel receptor‐specific vascular endothelial growth factor mutants. J Biol Chem. 2001;276:3222–3230. 10.1074/jbc.M002016200 11058584

[pro70529-bib-0024] He K , Zhang X , Ren S , Sun J . Deep residual learning for image recognition. Proc IEEE Comput Soc Conf Comput vis Pattern Recognit. Piscataway, NJ, USA: IEEE; 2016. p. 770–778.

[pro70529-bib-0025] Hicklin DJ , Ellis LM . Role of the vascular endothelial growth factor pathway in tumor growth and angiogenesis. J Clin Oncol. 2005;23:1011–1027. 15585754 10.1200/JCO.2005.06.081

[pro70529-bib-0026] Anon Wimasis Image Analysis. Available from: https://www.wimasis.com/

[pro70529-bib-0027] Huang J , Mackerell AD . CHARMM36 all‐atom additive protein force field: validation based on comparison to NMR data. J Comput Chem. 2013;34:2135–2145. Available from: https://pubmed.ncbi.nlm.nih.gov/23832629/ 23832629 10.1002/jcc.23354 PMC3800559

[pro70529-bib-0028] Justus CR , Marie MA , Sanderlin EJ , Yang LV . Transwell in vitro cell migration and invasion assays. Methods Mol Biol. 2023;2644:349–359. 37142933 10.1007/978-1-0716-3052-5_22 PMC10335869

[pro70529-bib-0029] Kastana P , Tuz Zahra F , Ntenekou D , Katraki‐Pavlou S , Beis D , Lionakis MS , et al. Matrigel plug assay for in vivo evaluation of angiogenesis. Methods Mol Biol. 2019;1952:219–232. 30825178 10.1007/978-1-4939-9133-4_18

[pro70529-bib-0030] Kendall RL , Thomas KA . Inhibition of vascular endothelial cell growth factor activity by an endogenously encoded soluble receptor. Proc Natl Acad Sci USA. 1993;90:10705–10709. 8248162 10.1073/pnas.90.22.10705 PMC47846

[pro70529-bib-0031] Khachigian LM , Liew G , Teo KYC , Wong TY , Mitchell P . Emerging therapeutic strategies for unmet need in neovascular age‐related macular degeneration. J Transl Med. 2023;21:1–17. 10.1186/s12967-023-03937-7 36810060 PMC9942398

[pro70529-bib-0032] Khurana R , Simons M , Martin JF , Zachary IC . Role of angiogenesis in cardiovascular disease: a critical appraisal. Circulation. 2005;112:1813–1824. 16172288 10.1161/CIRCULATIONAHA.105.535294

[pro70529-bib-0033] Kretschmer M , Rüdiger D , Zahler S . Mechanical aspects of angiogenesis. Cancers. 2021;13:4987. 34638470 10.3390/cancers13194987 PMC8508205

[pro70529-bib-0034] Lai L , Liu Y , Song B , Li K , Zeng X . Deep generative models for therapeutic peptide discovery: a comprehensive review. ACM Computing Surveys. 2025;57:1–29.

[pro70529-bib-0035] Martino MM , Tortelli F , Mochizuki M , Traub S , Ben‐David D , Kuhn GA , et al. Engineering the growth factor microenvironment with fibronectin domains to promote wound and bone tissue healing. Sci Transl Med. 2011;3:3. 10.1126/scitranslmed.3002614 21918106

[pro70529-bib-0036] Martinotti S , Ranzato E . Scratch wound healing assay. Methods Mol Biol. 2020;2109:225–229. 31414347 10.1007/7651_2019_259

[pro70529-bib-0037] Masson V , Devy L , Grignet‐Debrus C , Bernt S , Bajou K , Blacher S , et al. Mouse aortic ring assay: a new approach of the molecular genetics of angiogenesis. Biol Proced Online. 2002;4:24–31. 12734572 10.1251/bpo30 PMC145553

[pro70529-bib-0038] Meyer M , Clauss M , Lepple‐Wienhues A , Waltenberger J , Augustin HG , Ziche M , et al. A novel vascular endothelial growth factor encoded by Orf virus, VEGF‐E, mediates angiogenesis via signalling through VEGFR‐2 (KDR) but not VEGFR‐1 (Flt‐1) receptor tyrosine kinases. EMBO J. 1999;18:363–374. 9889193 10.1093/emboj/18.2.363 PMC1171131

[pro70529-bib-0039] Mosmann T . Rapid colorimetric assay for cellular growth and survival: application to proliferation and cytotoxicity assays. J Immunol Methods. 1983;65:55–63. 6606682 10.1016/0022-1759(83)90303-4

[pro70529-bib-0040] Okonkwo UA , Chen L , Ma D , Haywood VA , Barakat M , Urao N , et al. Compromised angiogenesis and vascular integrity in impaired diabetic wound healing. PLoS One. 2020;15:1–17. 10.1371/journal.pone.0231962 PMC7179900 32324828

[pro70529-bib-0041] Okonkwo UA , Dipietro LA . Diabetes and wound angiogenesis. Int J Mol Sci. 2017;18:1–15. 10.3390/ijms18071419 PMC5535911 28671607

[pro70529-bib-0042] Otrock ZK , Makarem JA , Shamseddine AI . Vascular endothelial growth factor family of ligands and receptors: review. Blood Cells Mol Dis. 2007;38:258–268. 17344076 10.1016/j.bcmd.2006.12.003

[pro70529-bib-0043] Papetti M , Herman IM . Mechanisms of normal and tumor‐derived angiogenesis. Am J Physiol—Cell Physiol. 2002;282:C947–C970. 11940508 10.1152/ajpcell.00389.2001

[pro70529-bib-0044] Rai V , Moellmer R , Agrawal DK . Stem cells and angiogenesis: implications and limitations in enhancing chronic diabetic foot ulcer healing. Cell. 2022;11:1–14. 10.3390/cells11152287 PMC9330772 35892584

[pro70529-bib-0045] Ramalho T , Filgueiras L , Silva‐Jr IA , Pessoa AFM , Jancar S . Impaired wound healing in type 1 diabetes is dependent on 5‐lipoxygenase products. Sci Rep. 2018;8:1–13. 30242286 10.1038/s41598-018-32589-7 PMC6155046

[pro70529-bib-0046] Rivilis I , Milkiewicz M , Boyd P , Goldstein J , Brown MD , Egginton S , et al. Differential involvement of MMP‐2 and VEGF during muscle stretch‐versus shear stress‐induced angiogenesis. Am J Physiol Heart Circ Physiol. 2002;283:1430–1438. 10.1152/ajpheart.00082.2002 12234794

[pro70529-bib-0047] Saaristo A , Tammela T , Farkkila A , Kärkkäinen M , Suominen E , Yla‐Herttuala S , et al. Vascular endothelial growth factor‐C accelerates diabetic wound healing. Am J Pathol. 2006;169:1080–1087. 16936280 10.2353/ajpath.2006.051251 PMC1698814

[pro70529-bib-0048] Santulli G , Ciccarelli M , Palumbo G , Campanile A , Galasso G , Ziaco B , et al. In vivo properties of the proangiogenic peptide QK. J Transl Med. 2009;7:1–10. 19505323 10.1186/1479-5876-7-41 PMC2702279

[pro70529-bib-0049] Shibuya M . Vascular endothelial growth factor (VEGF) and its receptor (VEGFR) signaling in angiogenesis: a crucial target for anti‐ and pro‐angiogenic therapies. Genes Cancer. 2011;2:1097–1105. 22866201 10.1177/1947601911423031 PMC3411125

[pro70529-bib-0050] Shibuya M . Vascular endothelial growth factor and its receptor system: physiological functions in angiogenesis and pathological roles in various diseases. J Biochem. 2013;153:13–19. 23172303 10.1093/jb/mvs136 PMC3528006

[pro70529-bib-0051] Staudemeyer RC , Morris ER . Understanding LSTM—a tutorial into Long Short‐Term Memory Recurrent Neural Networks. 2019 Available from: https://arxiv.org/abs/1909.09586v1

[pro70529-bib-0052] Tammela T , Alitalo K . Lymphangiogenesis: molecular mechanisms and future promise. Cell. 2010;140:460–476. 20178740 10.1016/j.cell.2010.01.045

[pro70529-bib-0053] Testa U , Pannitteri G , Condorelli GL . Vascular endothelial growth factors in cardiovascular medicine. J Cardiovasc Med. 2008;9:1190–1221. 10.2459/JCM.0b013e3283117d37 19001927

[pro70529-bib-0054] Tsai CY , Salawu EO , Li H , Lin GY , Kuo TY , Voon L , et al. Helical structure motifs made searchable for functional peptide design. Nat Commun. 2022;13:1–14. 35013238 10.1038/s41467-021-27655-0 PMC8748493

[pro70529-bib-0055] Wan F , Kontogiorgos‐Heintz D , de la Fuente Nunez C . Deep generative models for peptide design. Digit Discov. 2022;1:195–208. 35769205 10.1039/d1dd00024a PMC9189861

[pro70529-bib-0056] Wang R , Gu S , Kim YH , Lee A , Lin H , Jiang D . Diabetic wound repair: from mechanism to therapeutic opportunities. MedComm. 2025;6:e70406. 41030912 10.1002/mco2.70406 PMC12477442

[pro70529-bib-0057] Ware JA , Simons M . Angiogenesis in ischemic heart disease. Nat Med. 1997;3:158–164. 10.1038/nm0297-158 9018233

[pro70529-bib-0058] Yang S , Graham J , Kahn JW , Schwartz EA , Gerritsen ME . Functional roles for PECAM‐1 (CD31) and VE‐cadherin (CD144) in tube assembly and lumen formation in three‐dimensional collagen gels. Am J Pathol. 1999;155:887–895. 10.1016/S0002-9440(10)65188-7 10487846 PMC1866895

