# Supplementary material for: AI‐assisted design of a VEGFR2 agonistic peptide that promotes angiogenesis and wound repair
Source: Protein Sci. 2026 Mar 12;35(4):e70529. doi: 10.1002/pro.70529 (PMC13140732; doi:10.1002/pro.70529)
Supplement: Supplementary file 1 — Figure S1: Molecular dynamics (MD) simulations of the VEGFR2–VMP3 complex. Figure S2: Comparison of structural RMSD of VMP3 docked structure and final molecular dynamics structure. Figure S3: Comparison of structural RMSD of VMP3 crystal structure and final molecular dynamics structure. Figure S4: Comparison of structural RMSD of VMP3 bound VEGF‐R2 and apo form structure at 300 ns. Figure S5: RMSD of VEGF‐R2 during molecular dynamics simulation. Figure S6: PathScan ELISA analysis of VEGFR2 phosphorylation in response to VMP3 and a negative control peptide. Figure S7: Densitometric Analysis of Western Blot Results. Figure S8: Mouse Aortic Ring Assay (Whole section). Figure S9: Background noise deletion and sprout vessels boundaries detection. Figure S10: Sprout vessels area detection. Figure S11: Distribution of CD31‐positive cells across different treatment groups, at scale 60 μm. Figure S12: Whole‐slide images of wound tissues section. Figure S13: HPLC and MS graphs of VMP3 synthesis. Table S1: Physicochemical and experimental characterization of AI‐assisted VEGF‐mimetic peptides. Table S2: Molecular dynamics parameters implemented for MD production. Table S3: Binding free energy criteria. Table S4: Site‐directed mutagenesis of energetically favorable residues at VMP3 binding site. [file PRO-35-e70529-s001.docx]

***AI-assisted design of a VEGFR2 agonistic peptide that promotes angiogenesis and wound repair***

Farzana Yasmeen^a,b+^, Rajath Ramachandran^a,b+^, Rameez Hassan Pirzada^a+^, Bogeum Choi^a^, Hana Seo^a,b^, Wook Kim^a^, Moon Suk Kim^a*^, Sangdun Choi^a,b*^

*^a^Department of Molecular Science and Technology, Ajou University, Suwon 16499, Republic of Korea*

*^b^S&K Therapeutics, Suwon 16502, Republic of Korea*

^*^Corresponding authors:

MSK: moonskim@ajou.ac.kr, SC: sangdunchoi@ajou.ac.kr

^+^These authors contributed equally to this manuscript

**This file includes:**

1. **Supplementary Figures S1 to S13**

***2.3.1 Chemicals***

VEGF_165_ (Cat: 293-VE) and VEGFR2 Fc Chimera (Cat: 357-KD) proteins were purchased from R&D Systems. Mitomycin C (MMC) was obtained from Sigma-Aldrich (St. Louis, MO, USA). Antibodies against p-VEGFR2 ((Tyr1175) (19A10), #2478), t-VEGFR2 #2479, p-AKT (Ser473, #9271), t-ERK1/2 (#9102), p-p38 MAPK (Thr180/Tyr182, #9211), t-p38 MAPK, #9212, p-JNK (Thr183/Tyr185, #9251), t-JNK ( #9252), Cleaved caspase-3 (#9661), HIF-α (#36169), COX2 (#4842), p-FAK #3283), p-GSK-3 #9336), and p-PLCγ1 (#36721) were purchased from Cell Signaling Technology, whereas those against β-actin (# 47778) and p-ERK1/2 (#81492) were sourced from Santa Cruz Technology Inc. (Dallas, TX, USA). The anti-VEGFR2 antibody AF357 purchased from R&D Systems and used at 0.25 µg/ml. Matrigel basement membrane matrix (Cat: 354234) was purchased from Corning Inc. (Corning Life Sciences, Corning, NY, USA).

VMP3 was synthesized and purified using high-performance liquid chromatography (HPLC) by Peptron Co., Ltd. (Yuseong-gu, Daejeon, Republic of Korea). The purity and homogeneity of VMP3 were confirmed by analytical HPLC, which showed a single dominant peak at a retention time of 7.175 min, corresponding to >95% purity (Figure S1 a). The identity of the peptide was further confirmed using mass spectrometry (MS), which detected a major molecular ion at m/z 1811.7, consistent with the expected mass of VMP3 (Figure S1 b). QK, which was used as the positive control, was also synthesized by Peptron Co., Ltd. Unless otherwise specified, all other reagents were of analytical grade, used as received, and stored according to the manufacturer’s recommendations.


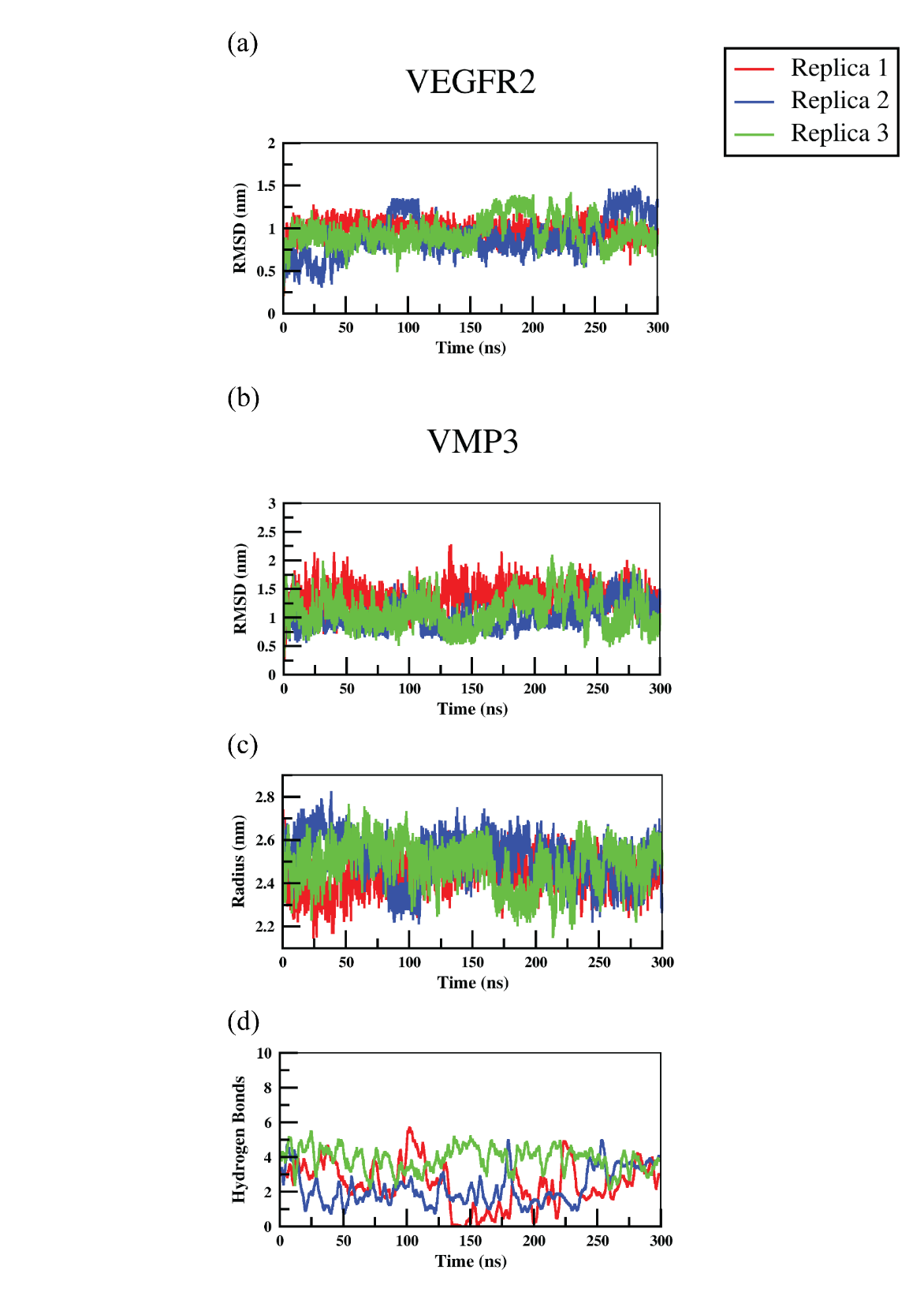


**Figure S1.** Molecular dynamics (MD) simulations of the VEGFR2-VMP3 complex. RMSD, radius of gyration, and hydrogen bond profiles are shown for VEGFR2–VMP3 over 300 ns in three independent simulations at different initial velocities. (a) RMSD of VEGFR2. (b) RMSD of VMP3 (c) Radius of gyration of VMP3-VEGFR2 complex backbone. (d) Average hydrogen bonds formed during simulation time. Consistent trends across replicas indicate stable and reproducible peptide-receptor interactions throughout the simulation period.


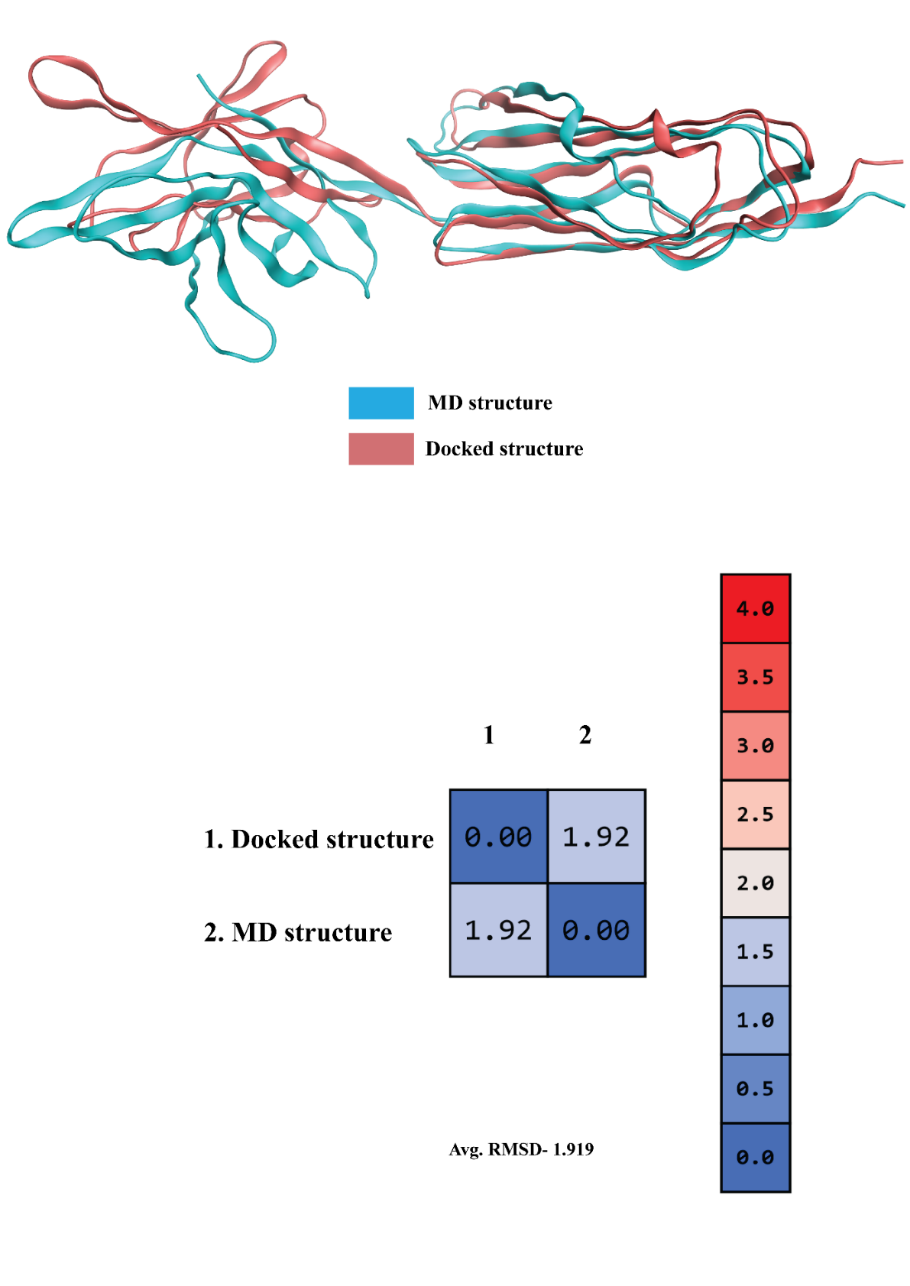


**Figure S2.** Comparison of structural RMSD of VMP3 docked structure and final molecular dynamics structure. The figure illustrates the structural variations in VEGFR2 after docking with VMP3 and in the final VMP3 bounded VEGFR2 structure sampled at 300 ns simulation.


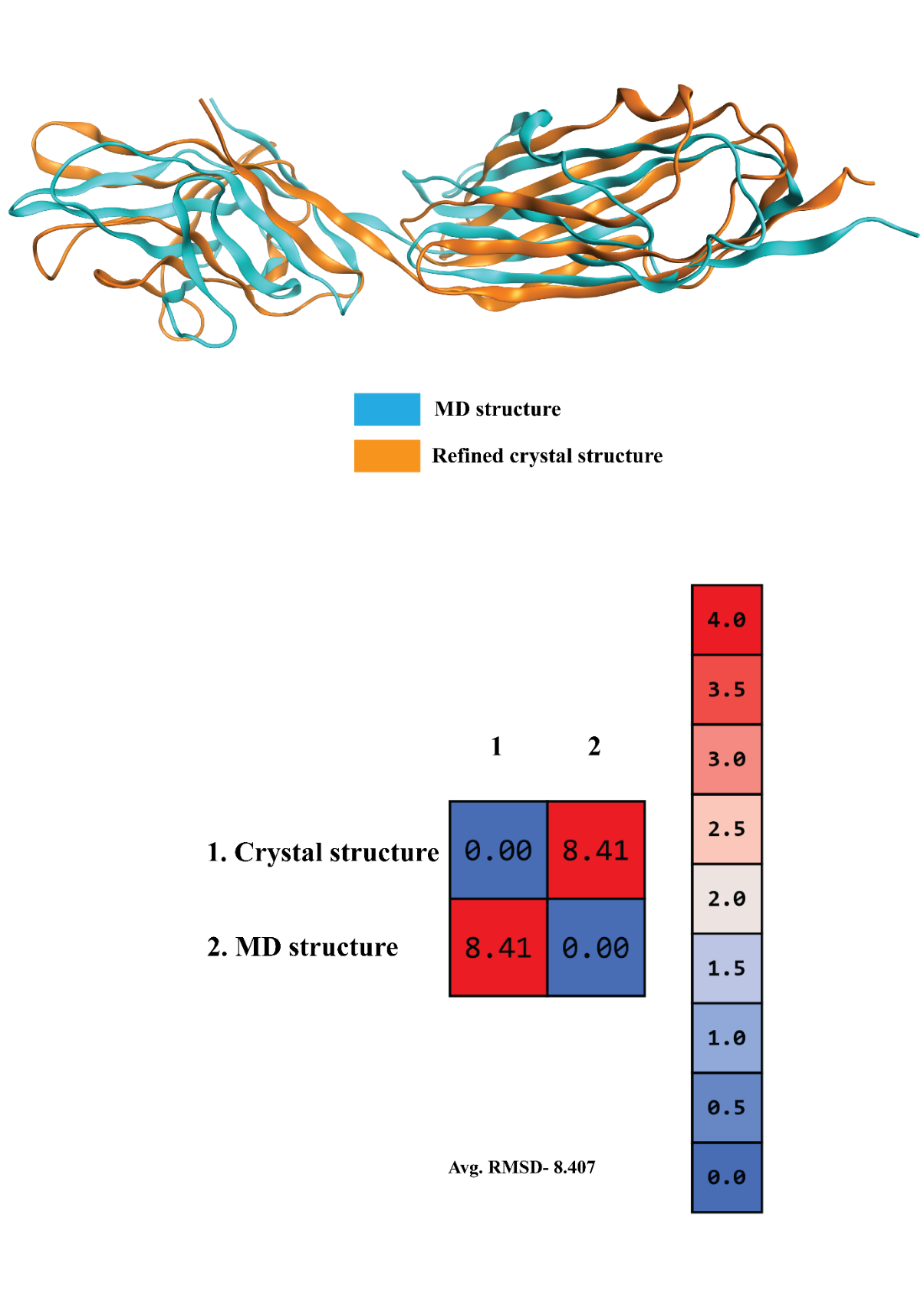


**Figure S3.** Comparison of structural RMSD of VMP3 crystal structure and final molecular dynamics structure. The superimposed structures of refined VEGFR2 structure and final VMP3 bounded VEGFR2 structure at 300 ns simulation time depicted here. A large RMSD value of 8.41 indicates the receptor underwent serious conformational changes that cause reorganization of its domains.


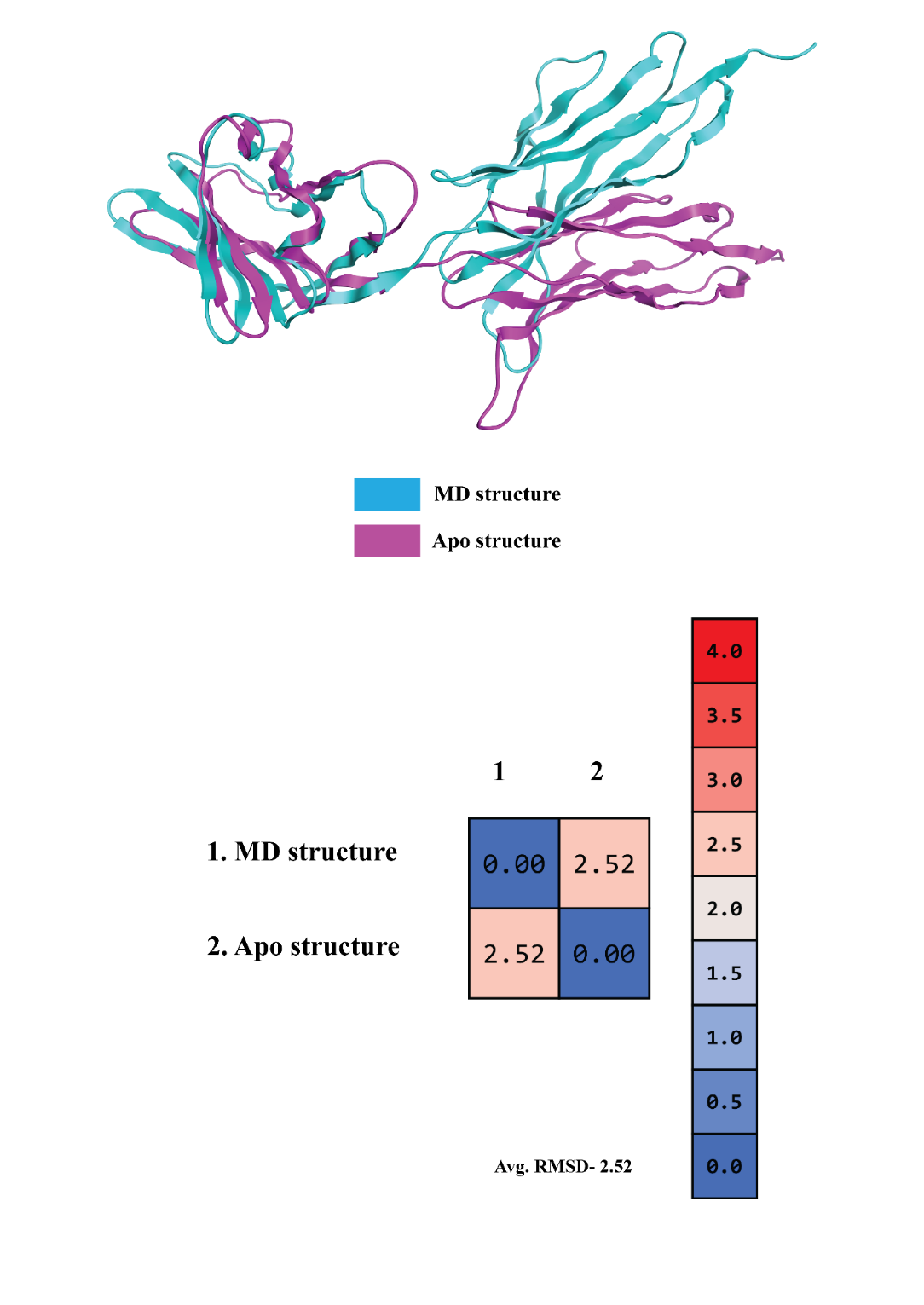


**Figure S4**: Comparison of structural RMSD of VMP3 bound VEGF-R2 and apo form structure at 300 ns. The figure illustrates the superimposed structures of VEGF-R2 in the absence (magenta) and presence (blue) of VMP3 at the 300 ns simulation time period. The domain 3 underwent serious conformational changes upon VMP3 binding with an RMSD of 2.52 Å compared to the final apo form structure.


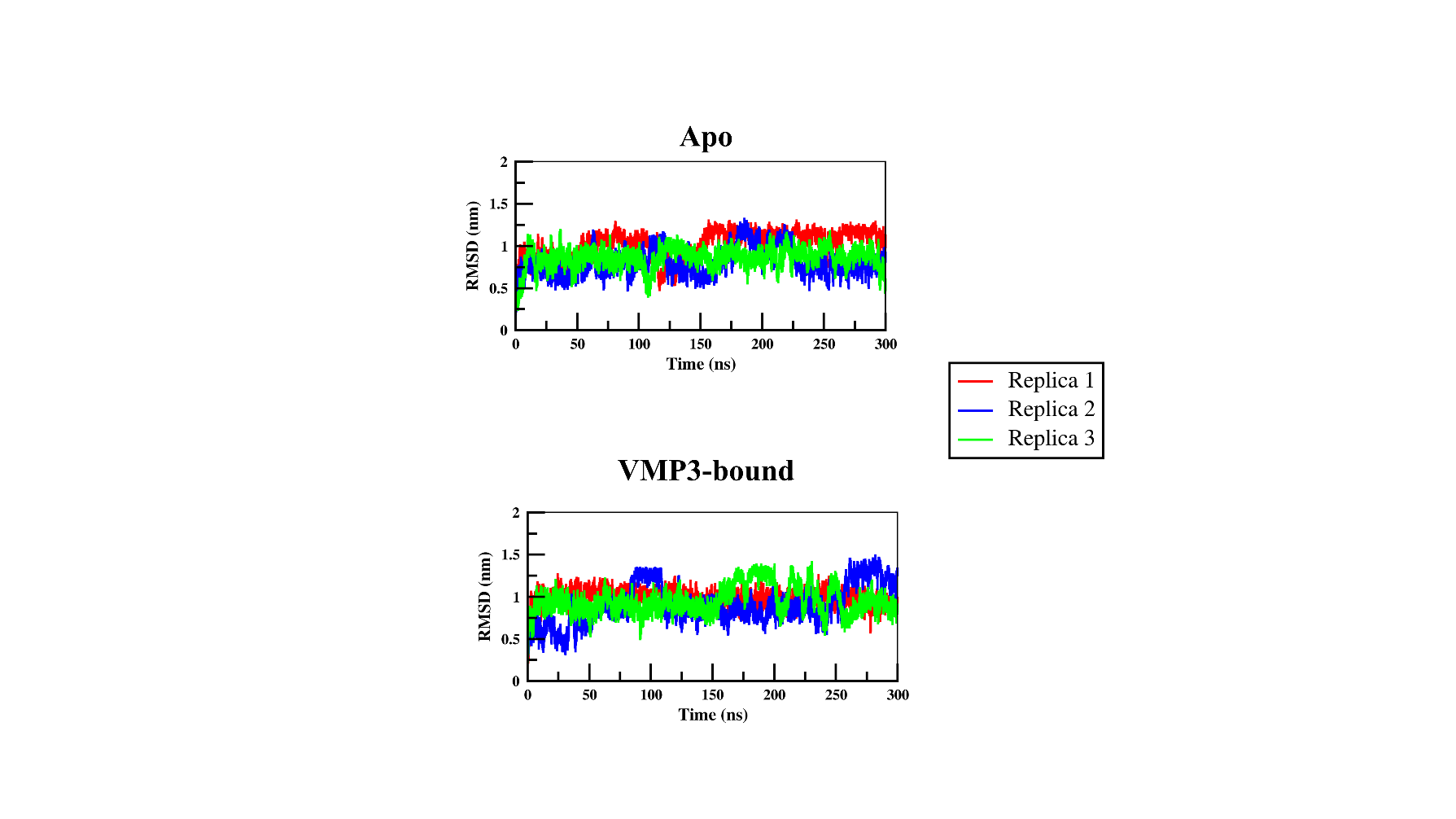


**Figure S5**: RMSD of VEGF-R2 during molecular dynamics simulation. The RMSD of VEGF-R2 during three independent molecular dynamics run as depicted namely, Apo and VMP3-bound. Apo form consists of native VEGF-R2, and the VMP3-bound system contains both VMP3 and VMGF-R2. A significant RMSD fluctuations were observed in VEGF-R2 backbone compared to the apo structure RMSD in the course of simulation.


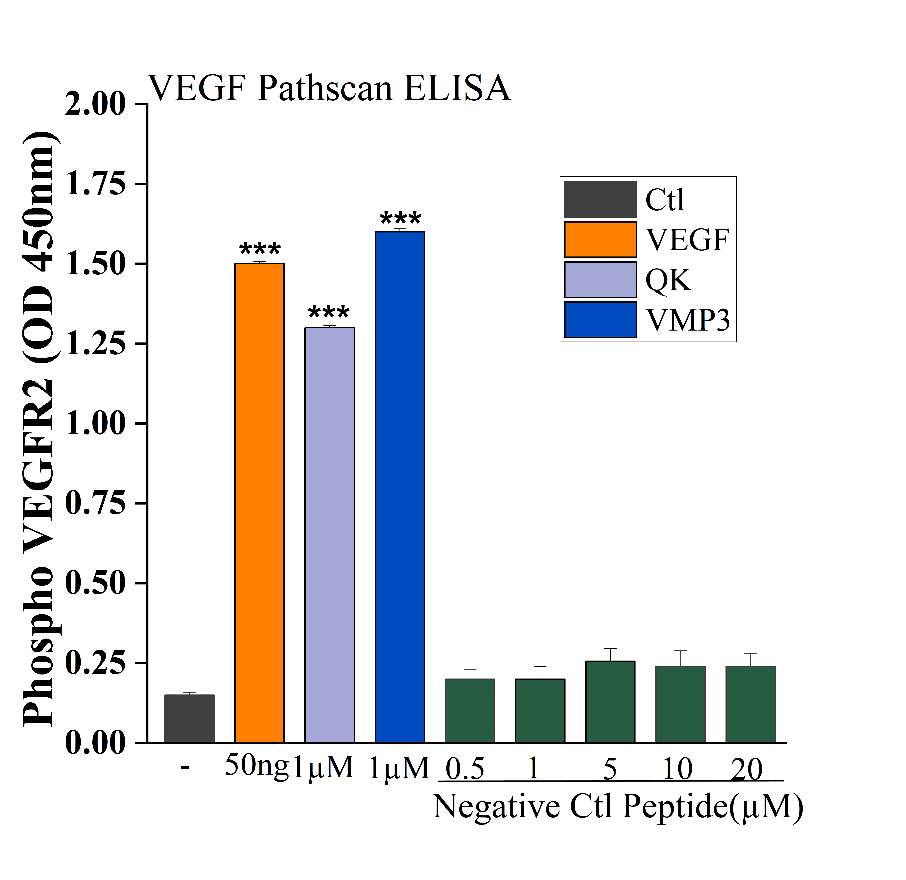


**Figure S6.** PathScan ELISA analysis of VEGFR2 phosphorylation in response to VMP3 and a negative control peptide. Phosphorylated VEGFR2 levels were quantified in HUVECs using a PathScan ELISA. Cells were treated with vehicle control (Ctl), VEGF (50 ng/mL), QK (1 μM), VMP3 (1 μM), or an inactive negative control peptide tested across a concentration range (0.5–20 μM). VEGF, QK, and VMP3 induced significant VEGFR2 phosphorylation, whereas the negative control peptide did not elicit VEGFR2 activation at any tested concentration, supporting the sequence-specific activity of VMP3. Data are presented as mean ± SD. ***p < 0.001 versus control.


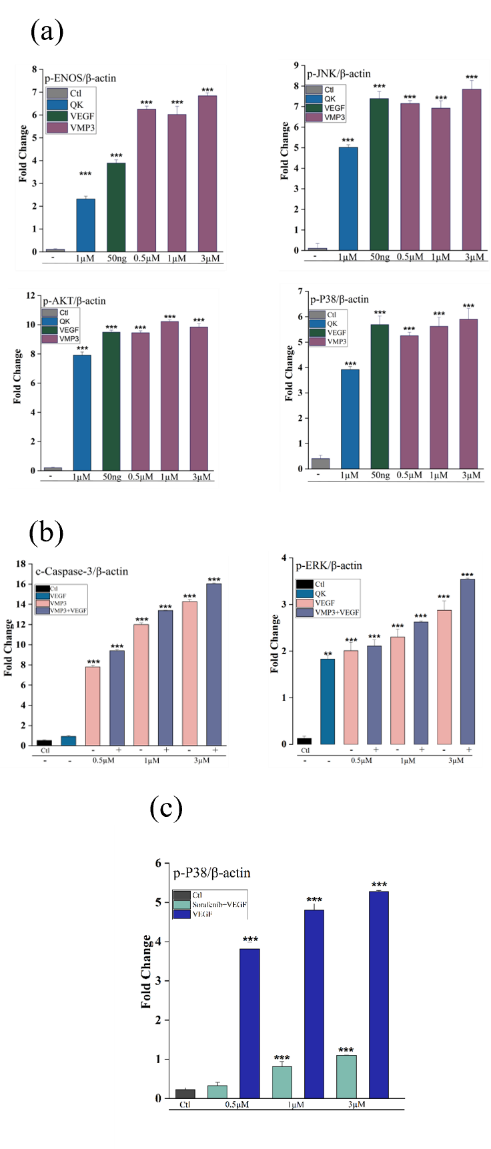


**Figure S7.** Densitometric Analysis of Western Blot Results. (a) only VMP3 peptide, (b) VMP3 peptide, with and without with VEGF ligand (c) VMP3 peptide with and without Sorafenib inhibitor.


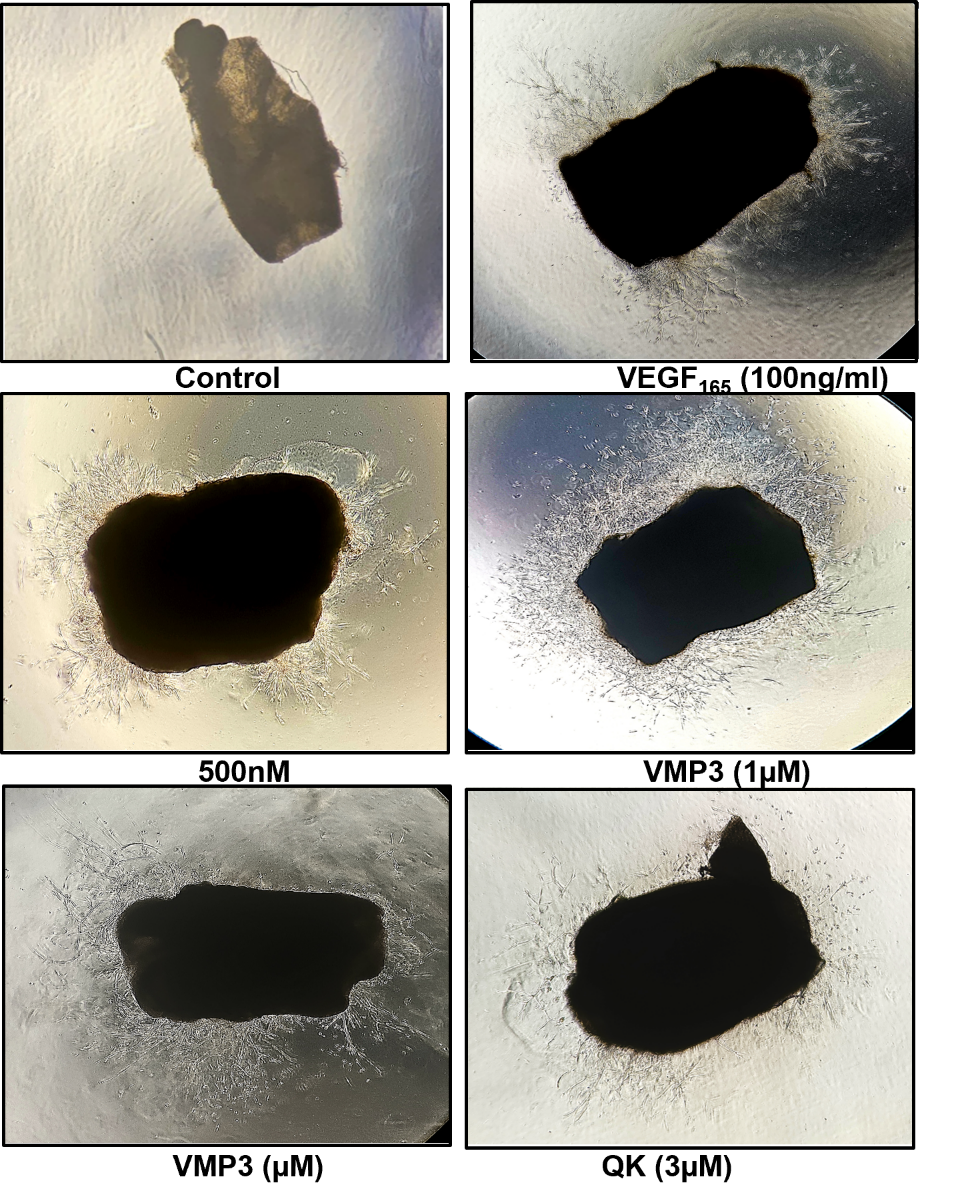


**Figure S8**. Mouse Aortic Ring Assay (Whole section)


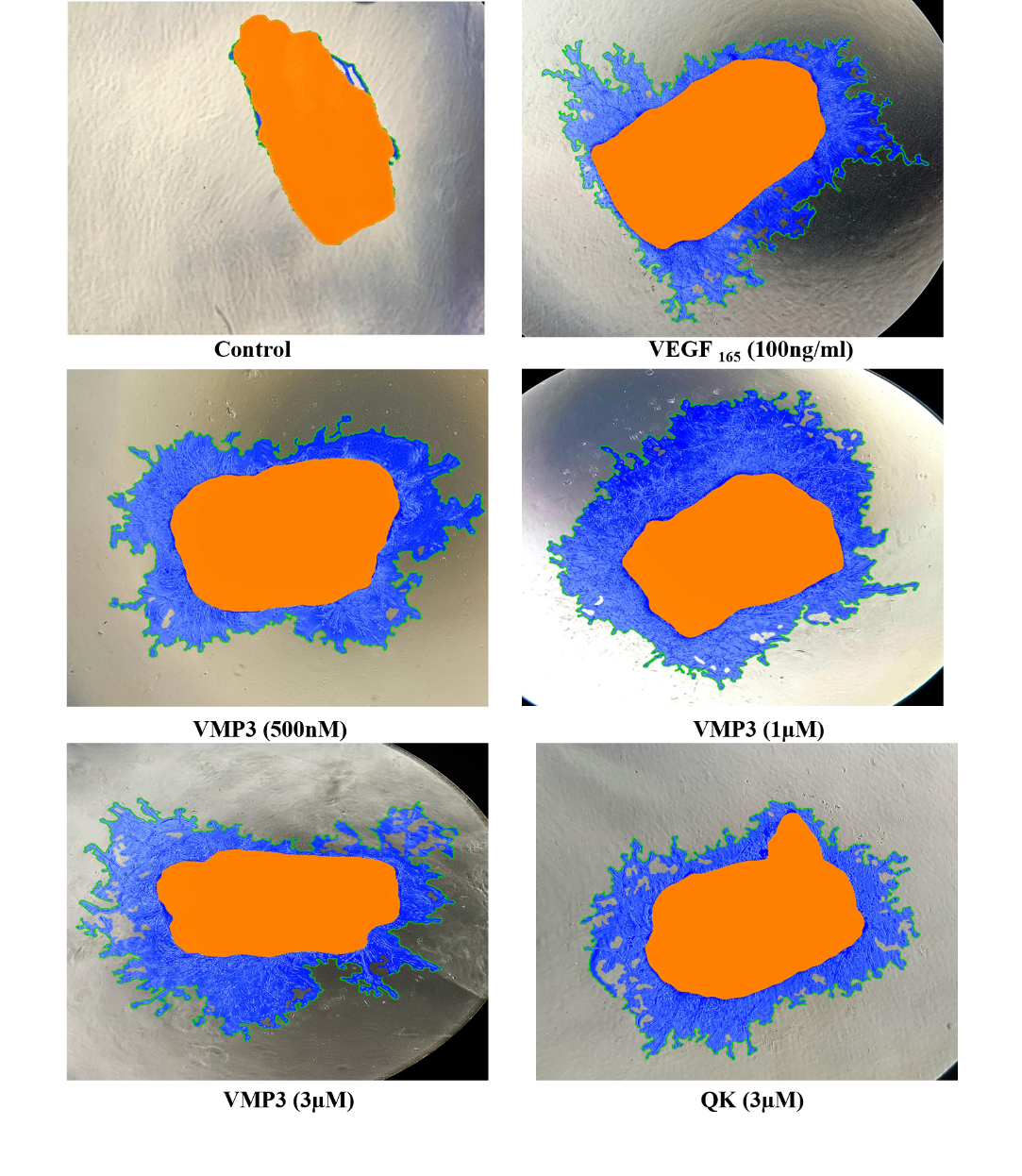


**Figure S9.** Background noise deletion and sprout vessels boundaries detection.

**
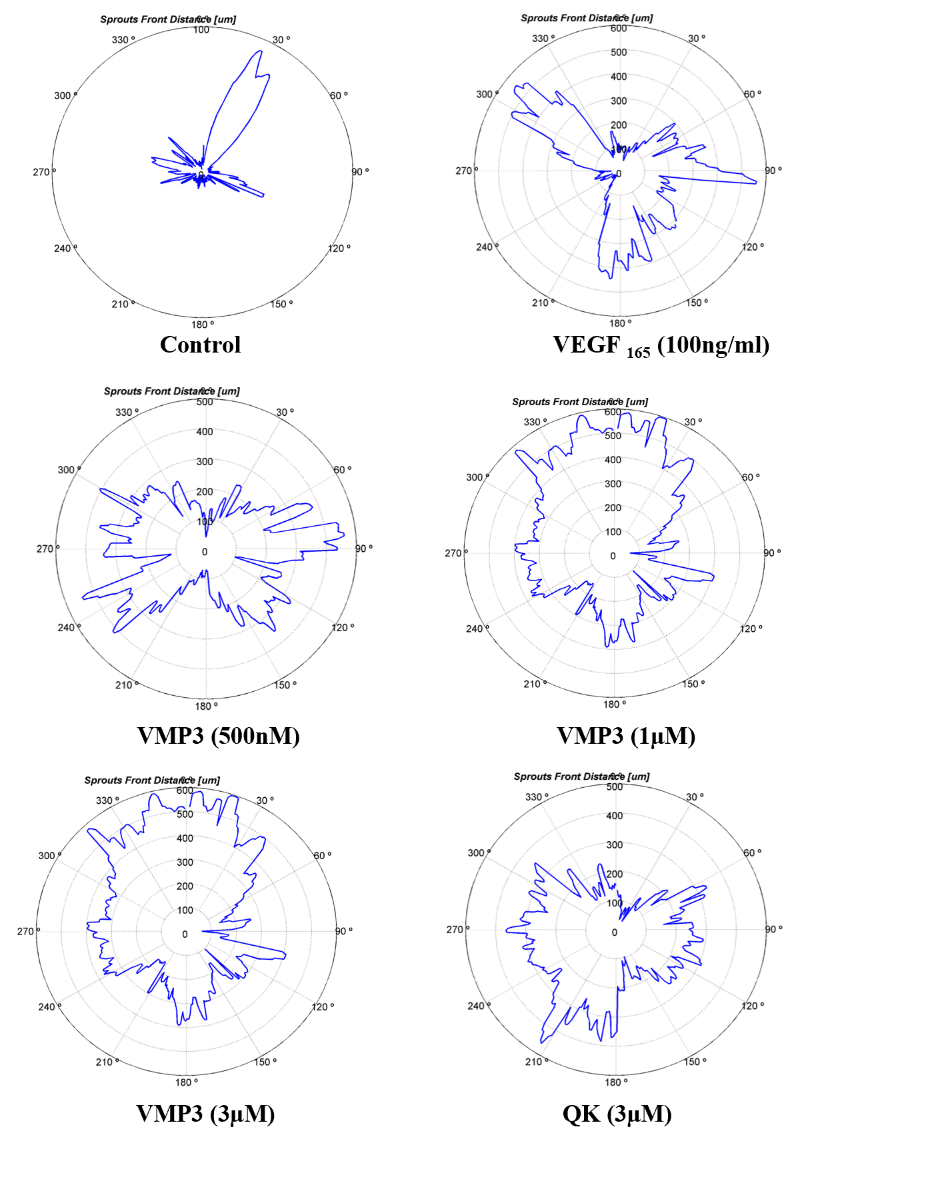
**

**Figure S10.** Sprout vessels area detection.

**
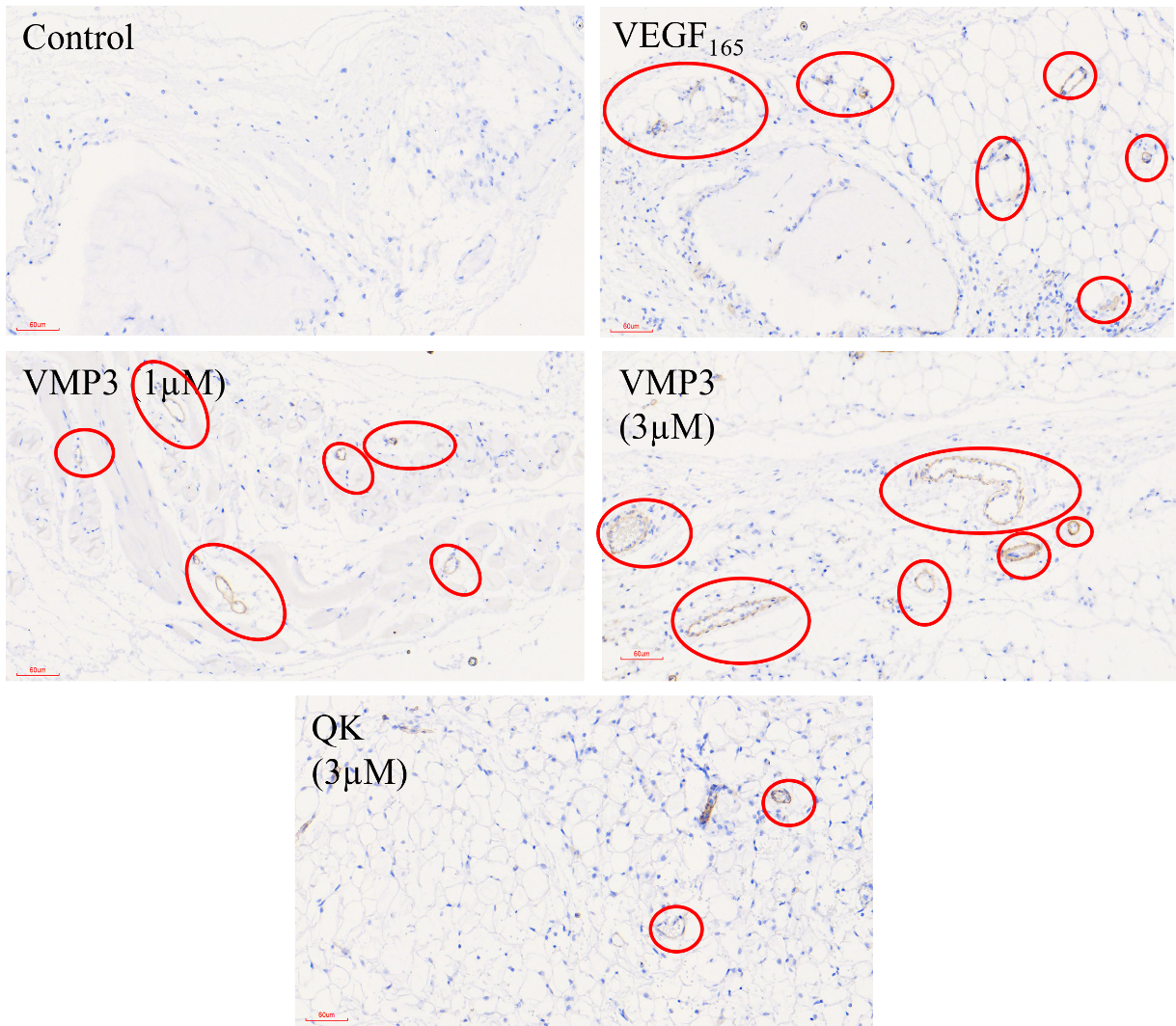
**

**Figure S11.** Distribution of CD31-positive cells across different treatment groups, at scale 60 µm.


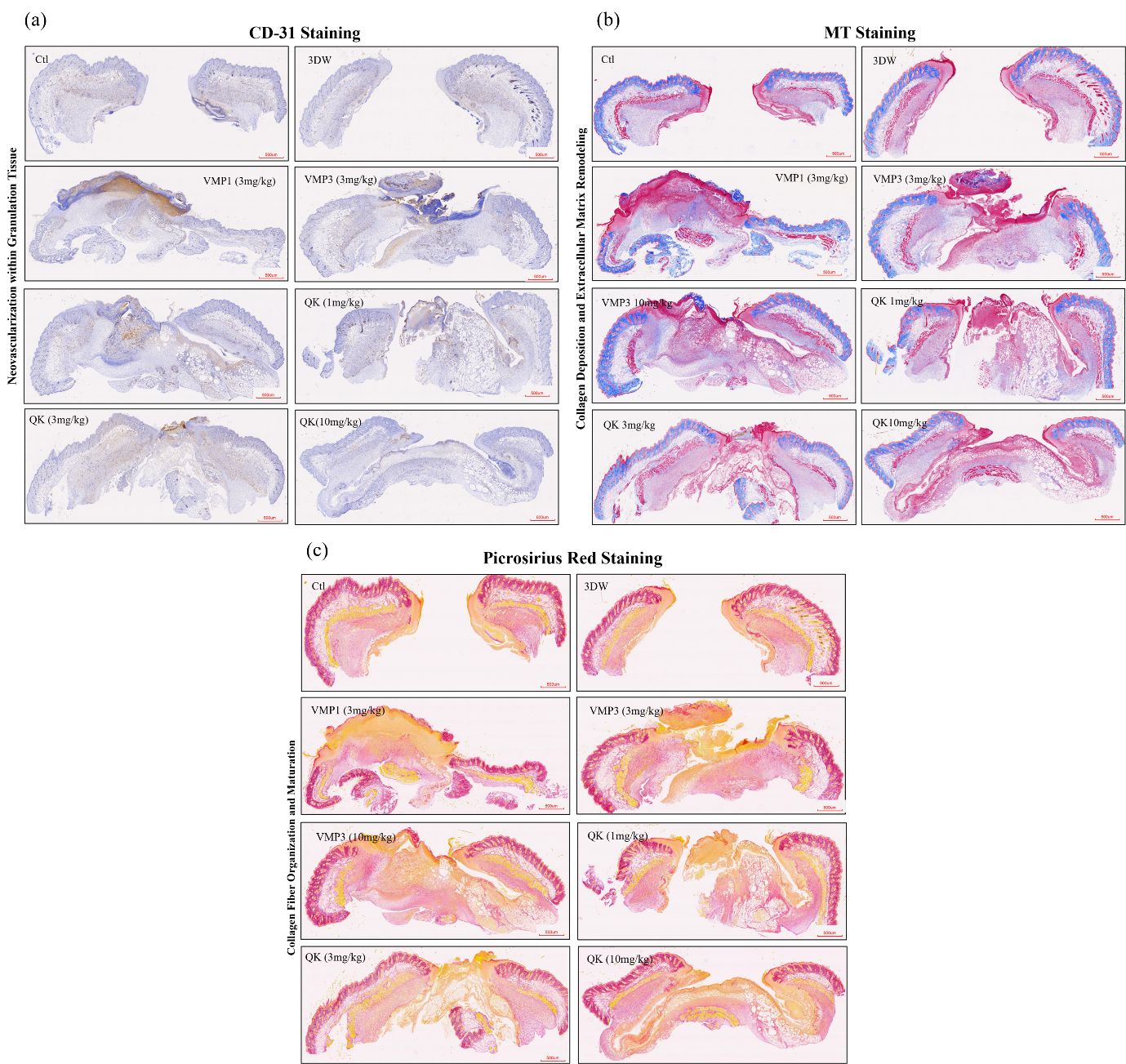


**Figure S12.** Whole-slide images of wound tissues section: stained with (a) CD31, (b) Masson’s Trichrome, and (c) Picrosirius Red (Day 5). Low-magnification (200 µm) views show the overall wound bed and surrounding dermis, corresponding to the high-magnification regions in Figure 8.


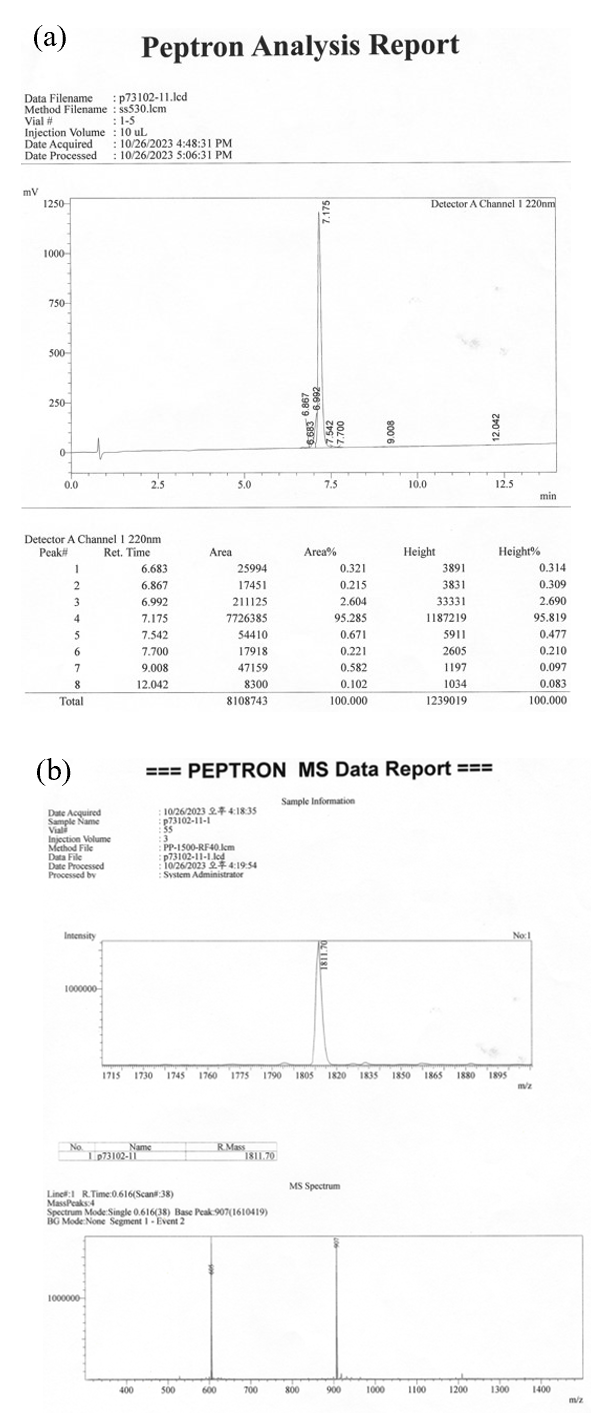


**Figure S13** HPLC and MS graphs of VMP3 synthesis. (a) High-performance liquid chromatography (HPLC) was employed to assess the purity and homogeneity of the synthesized peptide. The resulting chromatogram displays the peptide’s elution profile, with a prominent peak at a retention time of 7.175 minutes, indicative of a purity exceeding 95% (Peak 4). The area and height values for each peak are detailed in the accompanying table, where the major peak corresponds to the target peptide. The calculated purity of the peptide was 95.285%, reflecting the high quality of the synthesized compound. The analysis was conducted using a Shiseido capcell pak C18 column, with detection at 220 nm. (b) Mass spectrometric analysis of peptide sample p73102-11, as shown in the upper plot, presents the mass-to-charge ratio (m/z) against intensity, where a prominent peak at 1811.70 m/z corresponds to the peptide molecular ion. The lower spectrum indicates the base peak at 907 m/z. The observed variations in the mass spectrum fall within the expected limits for the sample, with no evidence of impurities detected.

**Supplementary Table S1 to S4**

**Table S1**: Physicochemical and experimental characterization of AI-assisted VEGF-mimetic peptides.

| Peptide ID | Amino acid sequence | Physicochemical properties | Levenshtein distance vs QK | In silico selection rationale | Experimental outcome |
| --- | --- | --- | --- | --- | --- |
| VMP1 | IHFMELYQATVCRP | L=13; Q=+1; pI=8.6; GRAVY=-0.1 | High | Helical propensity and hydrophobic–polar residue balance compatible with VEGF-mimetic design | No significant angiogenic activity |
| VMP2 | ARYMDGWNKSYTKP | L=13; Q=+1; pI=8.9; GRAVY=-0.4 | High | Sequence-divergent yet physiochemically permissible VEGF-like pattern | No significant angiogenic activity |
| VMP3 | ARFLEVWQRTYCKA | L=13; Q=+2; pI=9.31; GRAVY=0.2 | 12 (0.80) | Favorable balance of sequence novelty and amphipathic electrostatic features | Reproducible pro-angiogenic activity |
| VMP4 | AAMDVYQRSYCH | L=12; Q=+1; pI=8.3; GRAVY=-0.2 | High | Minimal-length helical scaffold compatible with VEGF-mimetic criteria | No significant angiogenic activity |
| VMP5 | VAFMDVYQRSYCAN | L=14; Q=+1; pI=8.7; GRAVY=0.1 | Moderate | Extended amphipathic character within acceptable physicochemical range | No significant angiogenic activity |
| VMP6 | PLFQFKPHNGQKL | L=12; Q=+2; pI=9.0; GRAVY=-0.5 | High | Electrostatically enriched sequence exploring AI-generated design diversity | No significant angiogenic activity |

**Note**: L = peptide length (amino acids); Q = net charge at physiological pH; pI = isoelectric point; GRAVY = grand average of hydropathy. All peptides were generated using an AI-assisted LSTM-based sequence model, refined using MOE-based structural evaluation, synthesized, and tested in identical in vitro angiogenesis assays. The VEGF-mimetic peptide QK was used solely as a reference for sequence similarity and physicochemical benchmarking.

**Table S2**: Molecular dynamics parameters implemented for MD production.

| **Molecular dynamics parameters** | |
| --- | --- |
| **Integrator** | Leap-frog integrator |
| **nsteps** | 150000000 |
| **nstlist** | 20 |
| **coulombtype** | Particle Mesh Ewald |
| **tcoupl** | V-rescale |
| **pcoupl** | Parrinello-Rahman |
| **cutoff-scheme** | Verlet |

**Table S3**: Binding free energy criteria.

| **MM/PBSA free energy calculation** | |
| --- | --- |
| **Frames** | 1000 |
| **Forcefield** | leaprc.protein.ff14SB |
| **istrng** | 0.15 |
| **fillratio** | 4 |
| **idecomp** | 2 |
| **dec_verbose** | 3 |
| **Distance cutoff** | 4 Å |

**Table S4**: Site-directed mutagenesis of energetically favorable residues at VMP3 binding site.

| **Mutation** | **Normal**  **(kcal/mol)** | **Mutant**  **(kcal/mol)** | **Mutant - Normal (ΔΔG)** |
| --- | --- | --- | --- |
| B:ILE:154/ ALA | -2.66 | - | 3.42 ± 2.18 |
| B:TYR:209/ ALA | -3.5 | 0.6 | 3.49 ± 1.06 |
